# Supplementary material for: Carboxy-terminal polyglutamylation regulates signaling and phase separation of the Dishevelled protein
Source: EMBO J. 2024 Sep 30;43(22):15. doi: 10.1038/s44318-024-00254-7 (PMC11574253; doi:10.1038/s44318-024-00254-7)
Supplement: Supplementary file 1 — Appendix [file 44318_2024_254_MOESM1_ESM.pdf]

Appendix for:

Carboxy-terminal polyglutamylation regulates signaling and phase separation of the Dishevelled protein

Marek Kravec, Ondrej Šedo, Jana Nedvědová, Miroslav Micka, Marie Šulcová, Nikodém Zezula, Kristína Gömöryová, David Potěšil, Ranjani Sri Ganji, Sara Bologna, Igor Červenka, Zbyněk Zdráhal, Jakub Harnoš, Konstantinos Tripsianes, Carsten Janke, Cyril Bařinka and Vítězslav Bryja*

*Corresponding author. Email: bryja@sci.muni.cz

This PDF file includes:

Appendix Fig. S1. 2
Appendix Fig. S2. 4
Appendix Fig. S3. 5
Appendix Fig. S4. 7
Appendix Fig. S5. 9

Appendix Figure S1

Standard C-term E10:

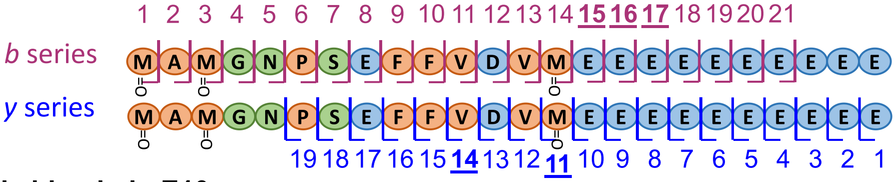

Standard side-chain E10:

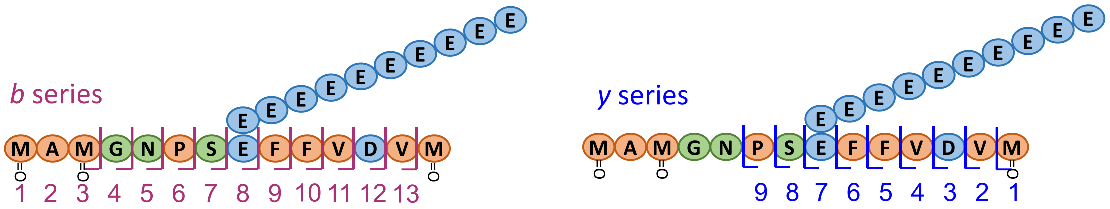

Sample DVL3 + TTLL11 :

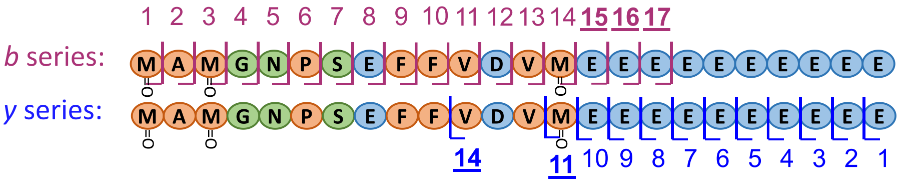

Standard: C-term E10, 3x M-ox

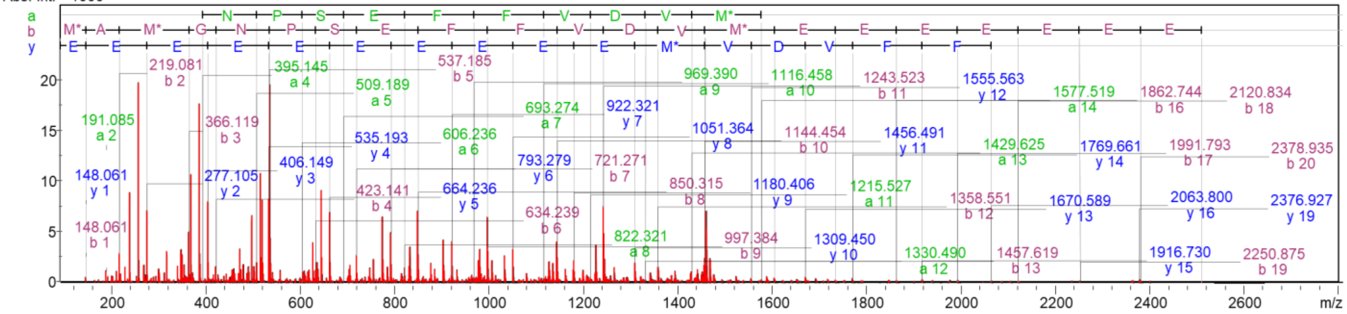

Standard: side-chain E10, 3x M-ox

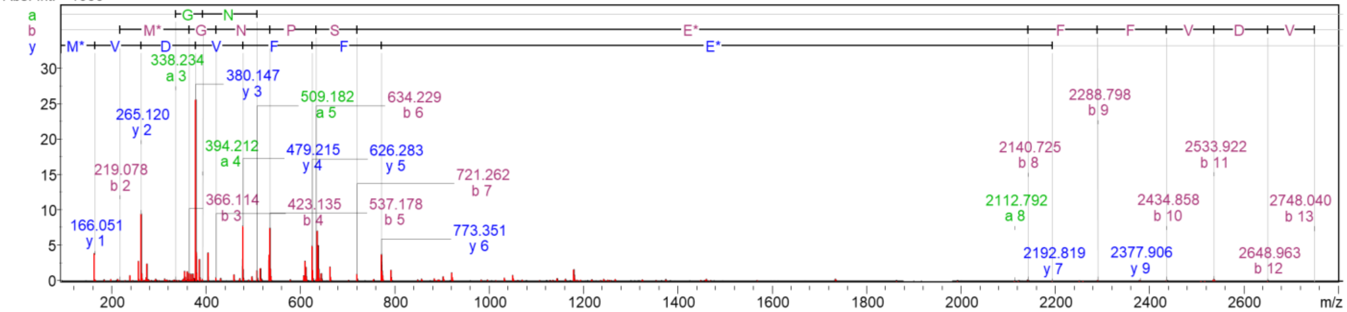

Sample: DVL3 WT + TTLL11, 3x M-ox (identical to Fig. 3D)

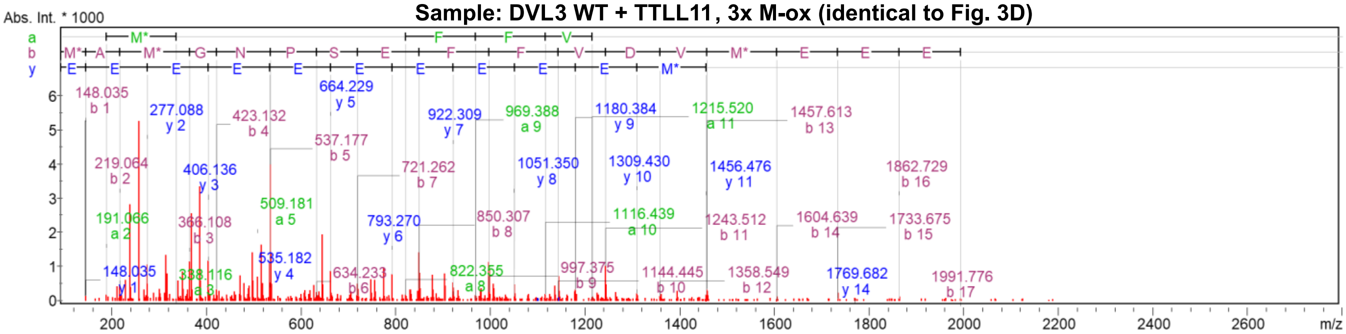

Appendix Fig. S1. A novel character of DVL3 PTM – MS/MS spectra.

MS/MS spectra of control synthetic peptides with 10E modification at M716 or E710 in comparison to the polyglutamylated DVL3 immunoprecipitated from cells. The aa sequences for individual peaks a shown in schematic representation above individual peaks in B and Y series. The fragments corresponding to modification on E710 are detected only in synthetic peptide control but not in DVL3 WT sample.

Appendix Figure S2

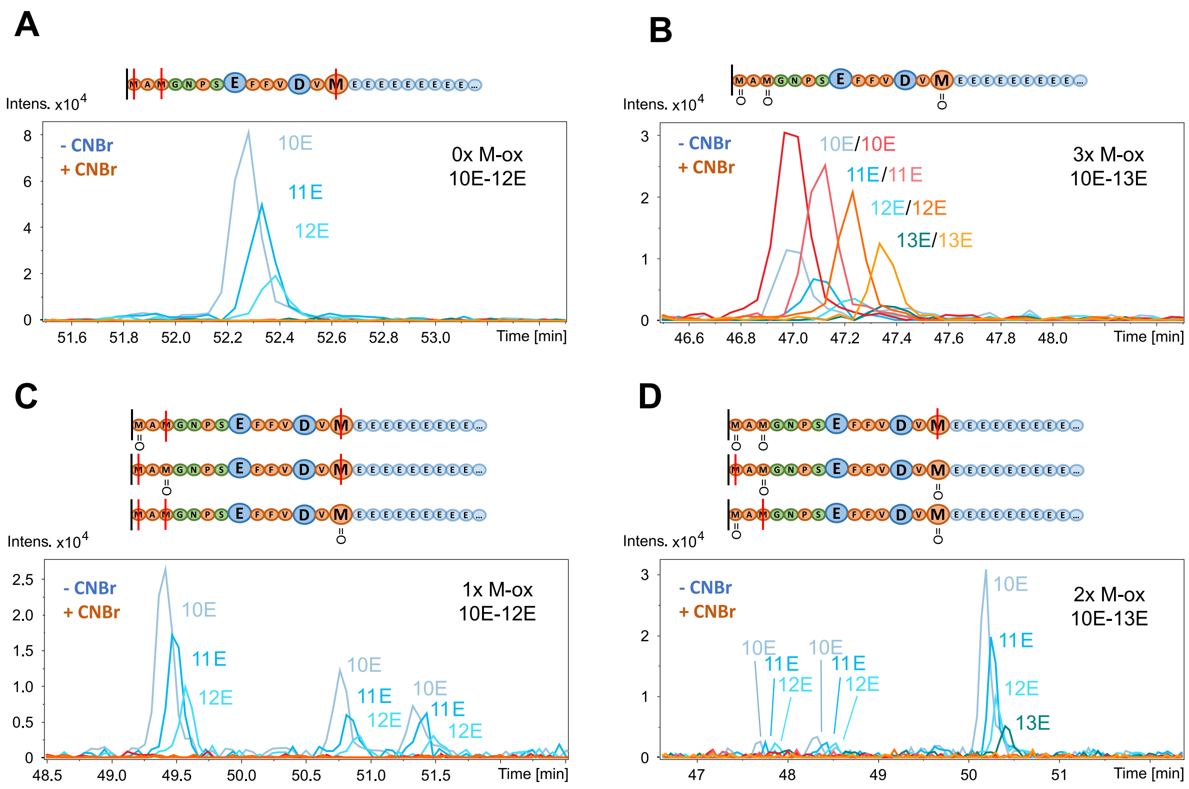

Appendix Fig. S2. A novel character of DVL3 PTM – CNBr cleavage.

(**A-D**) CNBr cleavage of polyglutamylated DVL3 C-terminal peptide in all its M-ox forms. EIC shows peaks corresponding to polyglutamylated peptides shown schematically above each chromatogram. Red line indicates cleavage site. A, C and D shows 0x, 1x and 2x M-ox peptides respectively, where all peptides were cleaved by CNBr. B chromatogram shows 3x M-ox peptide that cannot be cleaved by CNBr.

Appendix Figure S3

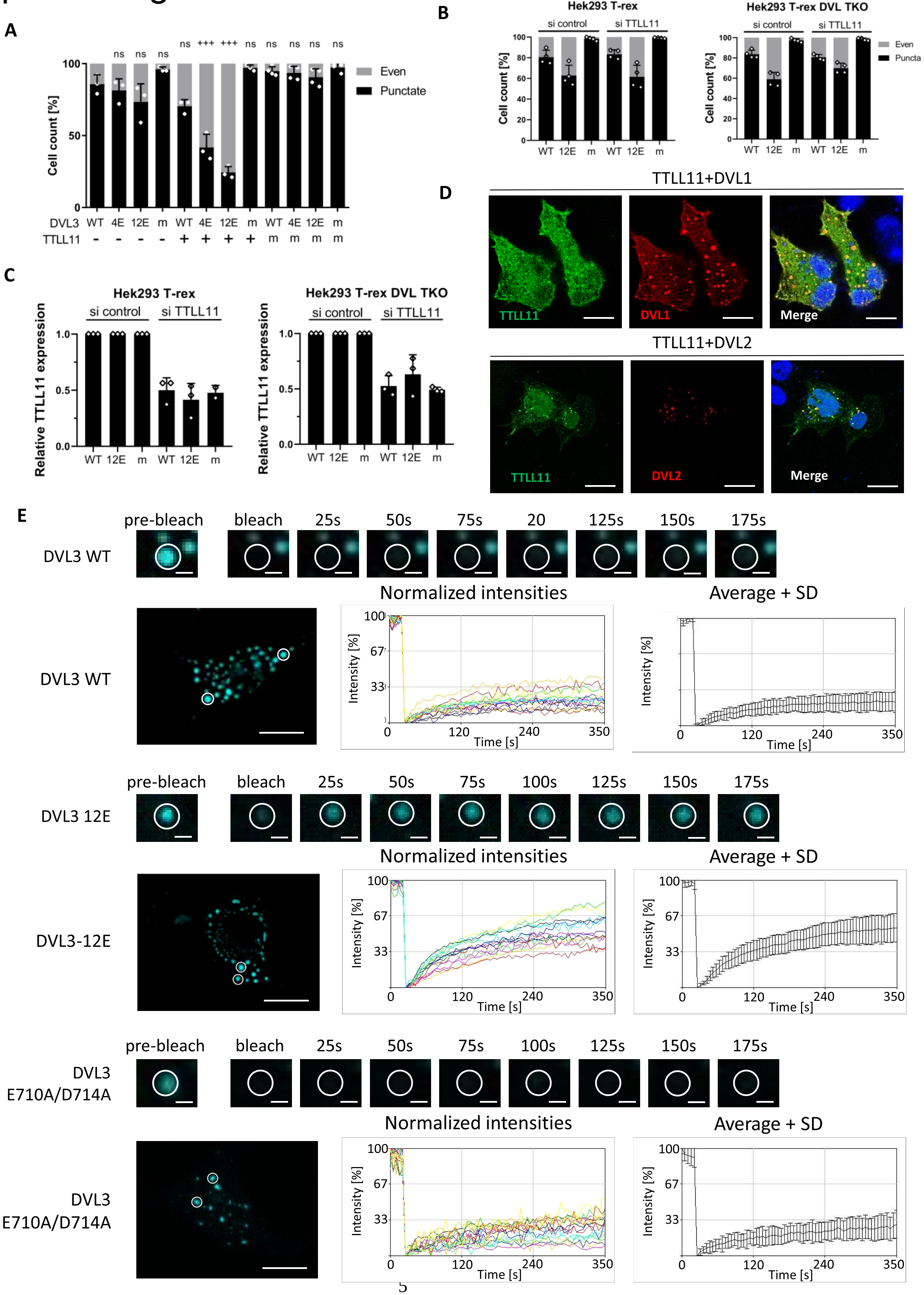

Appendix Figure S3. The effects of DVL3 polyglutamylation on the DVL3 phase separation. Analysis of punctate vs. even subcellular localization of overexpressed DVL3 and its polyglutamylated variant or DVL3m = DVL3 E710A/D714A in (**A**) with co-expressed TTLL11 in HEK293T cells or (**B**) with or without TTLL11 downregulation by siRNA in HEK293T T-rex WT or HEK293T T-rex DVL1-3 triple KO (TKO) cells. DVL3 localization (A, B) was assessed for at least 200 cells in at least three biological replicates. SD and individual data points (white dots) are indicated. Statistically significant difference from WT DVL3 (* - all not significant, n.s.) and from the same DVL variant in the absence of TTLL11 (+) is indicated. (**C**) Quantitative PCR analysis of TTLL11 expression (Normalized on Rps13 housekeeping gene) in the samples from B. (**D**) Co - localization of overexpressed DVL1 and DVL2 with TTLL11 in HEK293T cells. TTLL11 co-localizes with DVL1 and DVL2 in puncta. Scale bar = 10 μm. (**E**) FRAP analysis of DVL3 wt, DVL3-12E and DVL3 E710A/D714A intracellular condensates. The data represent normalized intensities and average + SD from 15 condensates measured in 3 biological replicates (5 condensates per replicate).

Appendix Figure S4

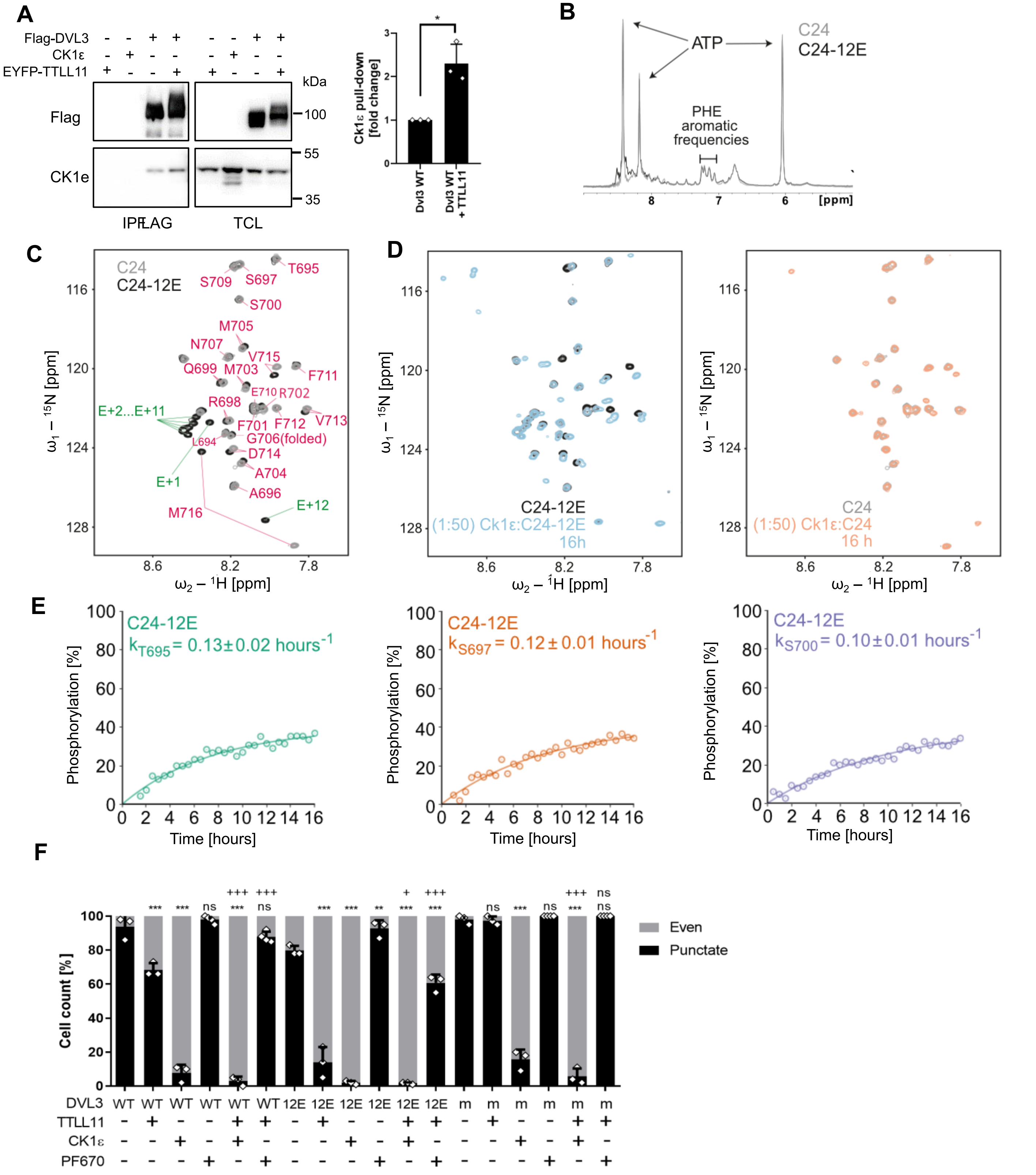

Appendix Fig. S4. The effect of polyglutamylation on the DVL3 phosphorylation by CK1ε.

(**A**) Co-immunoprecipitation of overexpressed DVL3 with endogenous CK1ε in presence and absence of co-expressed TTLL11 in HEK293T cells. The cells were transfected as indicated and pull-down was performed using specific antibodies. The graph quantifies the interaction between DVL3 and CK1ε upon co-expression of TTLL11. CK1ε band intensity was normalized to Flag intensity and results are represented as a fold change to CK1ε pull-down by DVL3 WT. Statistical significance was analyzed by one-sample t-test with theoretical mean = 1; n=3. (**B**) For real-time NMR phosphorylation reactions, the concentration of DVL3 C-terminal C24 and C24-12E peptides was matched in the aromatic frequencies of phenylalanine residues in 1D spectra. (**C**) Chemical shift assignment of C24 (grey) and C24-12E (black) peptides. (**D**) Overlay of HSQC spectra of non-phosphorylated form (grey and black for C24 and C24-12E, respectively) and phosphorylated form of the peptide (red and blue for C24 and C24-12E, respectively) after 16 h of the phosphorylation reaction. (**E**) Fraction of phosphorylated T695, S697 and S700 of C24-12E peptide over time. Rate constants were estimated from the mono-exponential fitting to experimental data. (**F**) Analysis of punctate vs. even subcellular localization of overexpressed DVL3 and its polyglutamylation variants (DVL3m = DVL3 E710A/D714A) upon co-expression or inhibition of CK1ε by PF670462 (1μM) in HEK293T cells. DVL3 localization was assessed for at least 200 cells in at least three biological replicates. SD and individual data points (white dots) are indicated. Statistically significant difference from the same DVL variant in the persence of TTLL11 (*) and from the same DVL variant in the presence of TTLL11 (+) is indicated. */+ represents $p < 0.05$; **/++ $p < 0.01$; ***/+++ $p < 0.001$; ns - not significant.

Appendix Figure S5

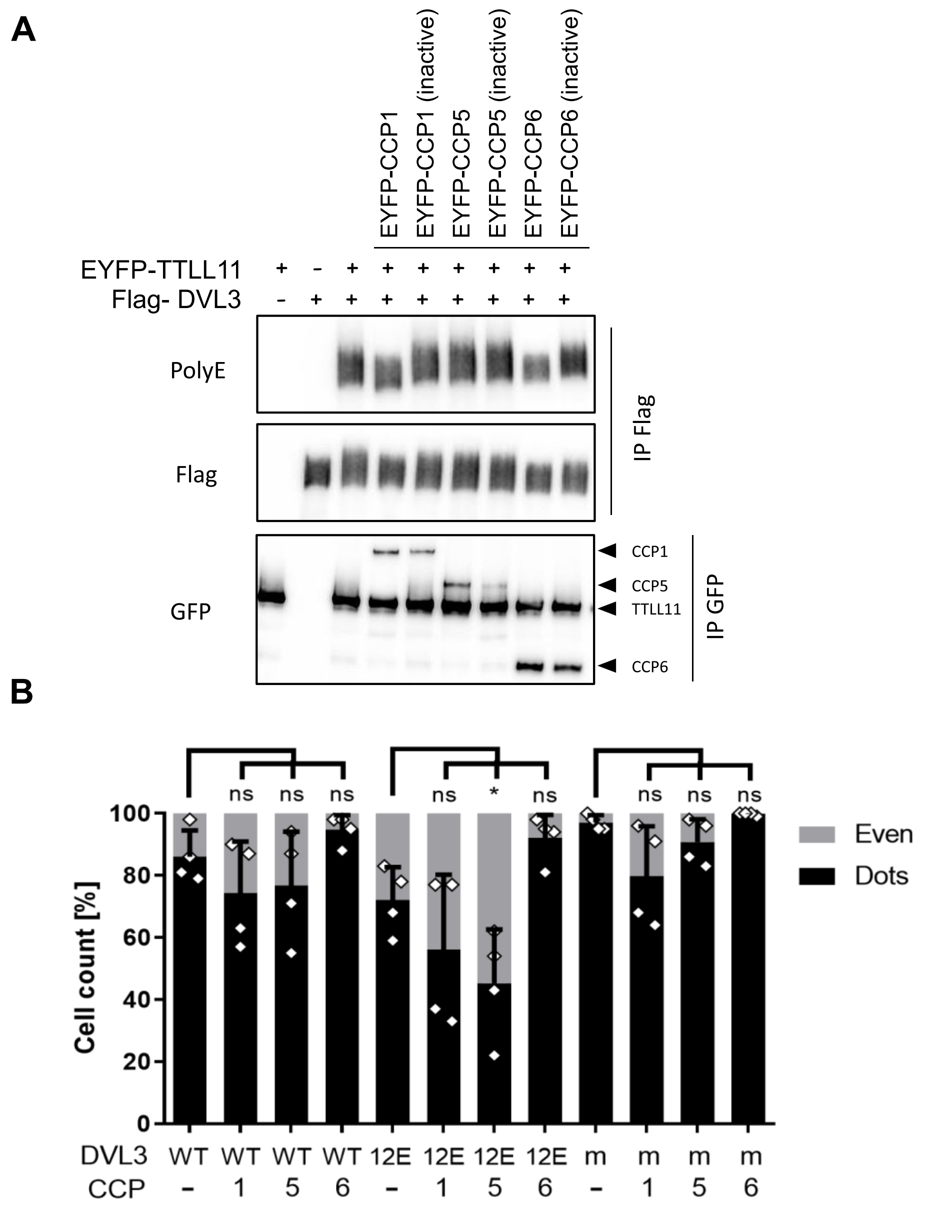

Appendix Fig. S5. Deglutamylation of DVL3 by CCP enzymes.

(**A**) DVL3 was co-expressed with EYFP-TTLL11 and wild type or inactive EYFP tagged CCP1, CCP5 and CCP6 in HEK293T cells. Upon lysis and immunoprecipitation of DVL3 the level of polyglutamylation was detected using modification-specific antibody PolyE in DVL3 pull-down (IP Flag). The level of CCPs and TTLL11 was monitored in the anti-GFP immunoprecipitates. The same co-IP of DVL3 and TTLL11 (first 3 lanes) is also shown in Figure 1D. (**B**) DVL3 variants (DVL3 m stands for E710A/D714A mutant) were co-expressed with EYFP-tagged CCP1, CCP5 and CCP6 in HEK293T cells and subcellular localization of DVL3 was analyzed by immunocytochemistry. Subcellular localization of DVL3 was assessed for at least 200 cells in 3 biological replicates. Mean + SD is shown. Statistical significance in comparison to the condition without CCP for each DVL3 variant is shown. * represents $p < 0.05$; ns - not significant.
