# Supplementary material for: Carboxy-terminal polyglutamylation regulates signaling and phase separation of the Dishevelled protein
Source: EMBO J. 2024 Sep 30;43(22):15. doi: 10.1038/s44318-024-00254-7 (PMC11574253; doi:10.1038/s44318-024-00254-7)
Supplement: Supplementary file 11 — Source data Fig. 5 [file 44318_2024_254_MOESM11_ESM.zip › 116133R_source data Fig 5/Source mircographs Fig. 5F.pptx]

## Slide 1
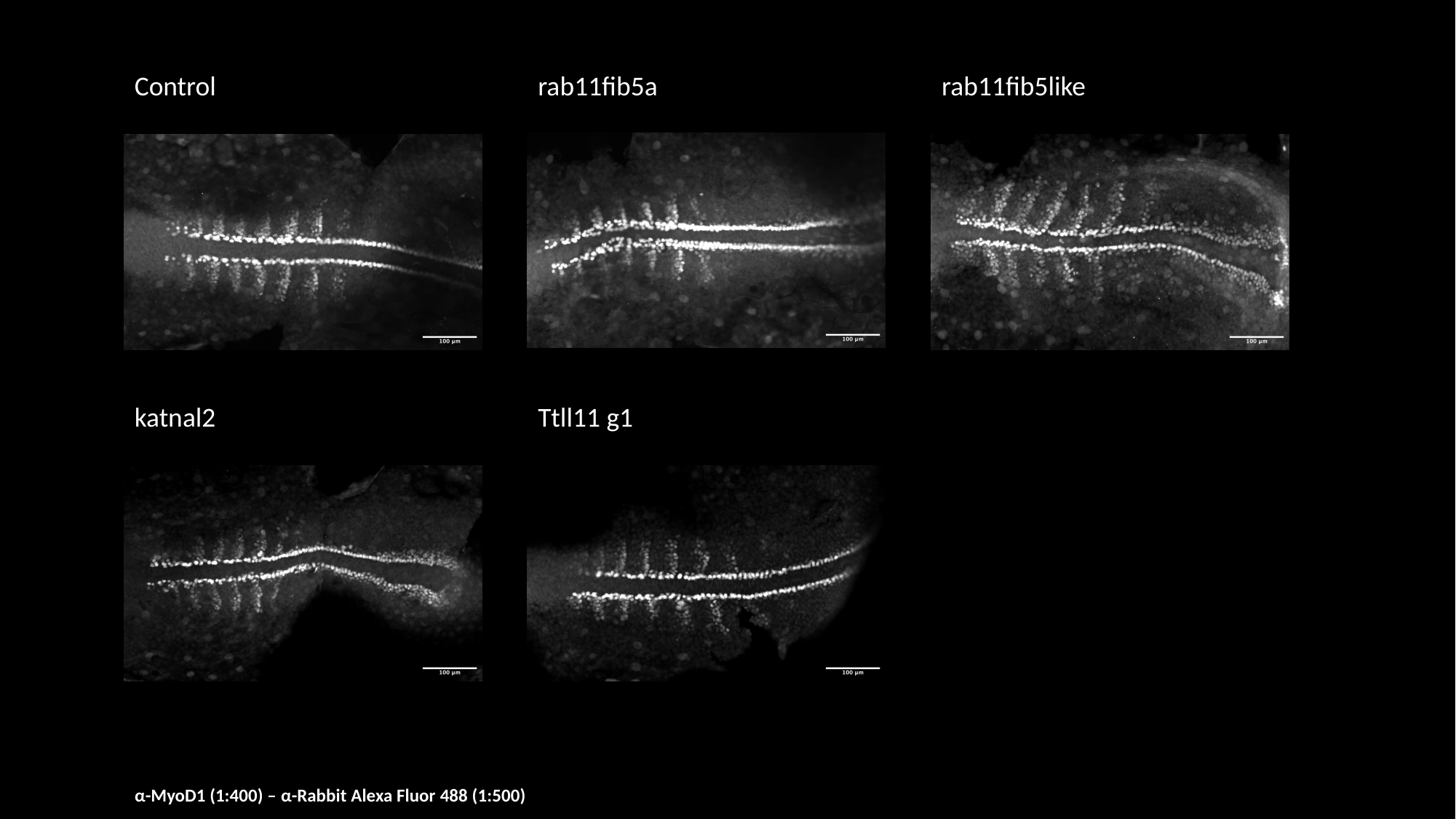

Control
rab11fib5a
rab11fib5like
katnal2
Ttll11 g1
α-MyoD1 (1:400) – α-Rabbit Alexa Fluor 488 (1:500)

## Slide 2
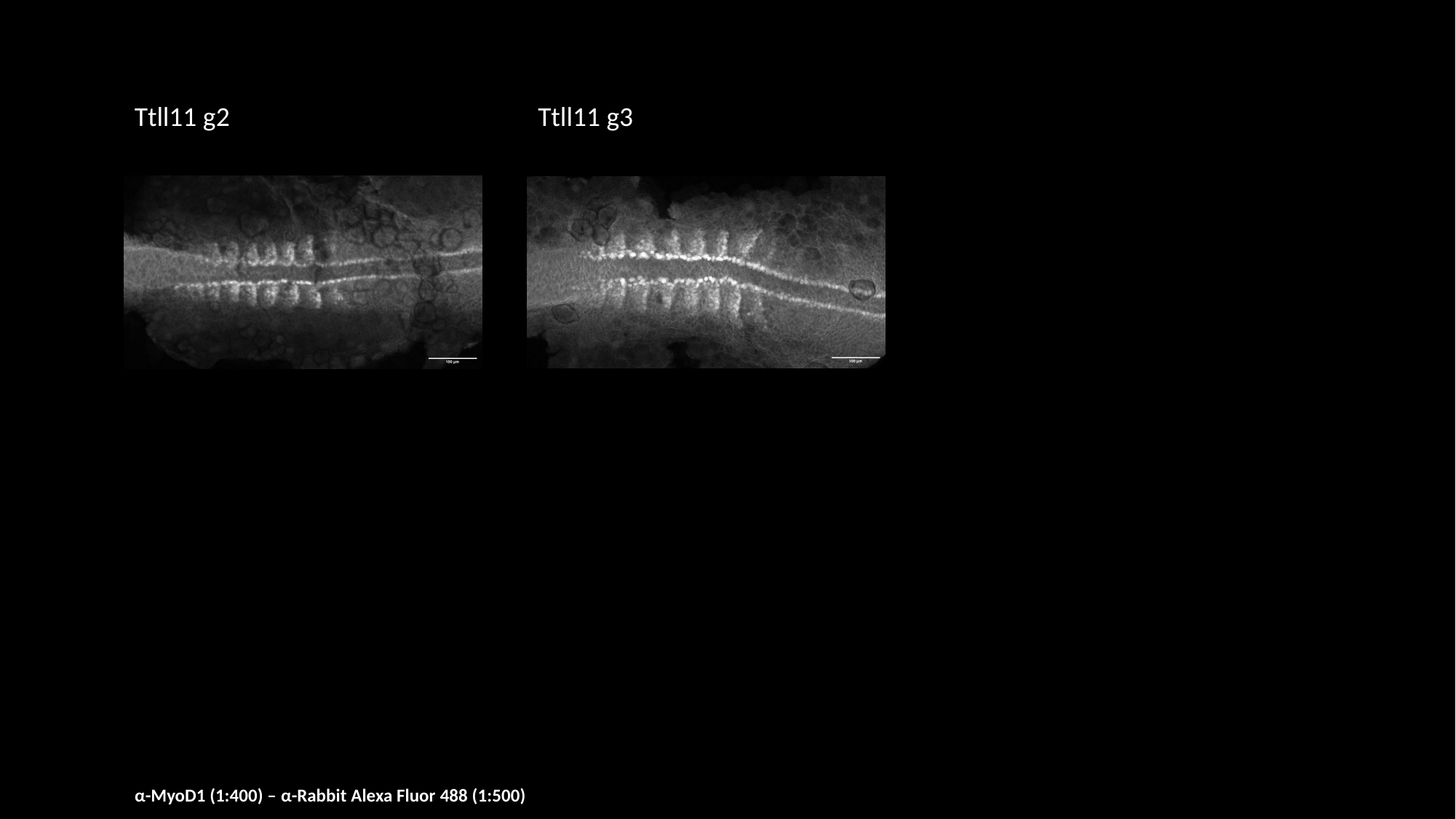

Ttll11 g2
Ttll11 g3
α-MyoD1 (1:400) – α-Rabbit Alexa Fluor 488 (1:500)
