# Supplementary figures and images for: Carboxy-terminal polyglutamylation regulates signaling and phase separation of the Dishevelled protein
Source: EMBO J. 2024 Sep 30;43(22):15. doi: 10.1038/s44318-024-00254-7 (PMC11574253; doi:10.1038/s44318-024-00254-7)

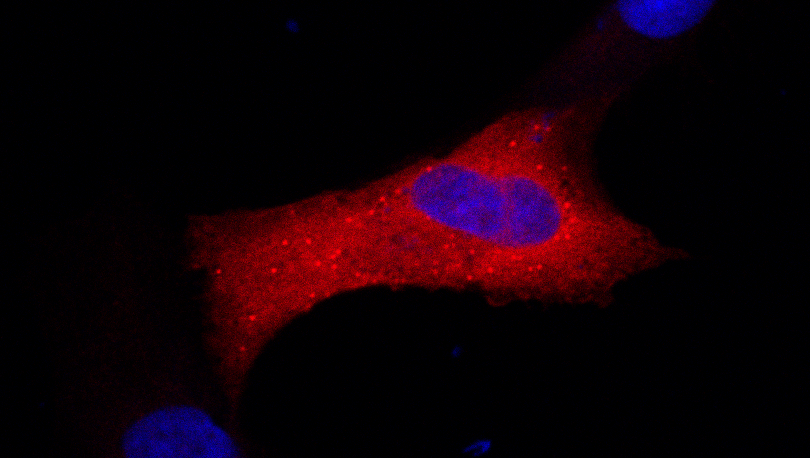

Supplement: Supplementary file 12 — Source data Fig. 6 [file 44318_2024_254_MOESM12_ESM.zip › 116133R_source data Fig 6/Micrographs/FIg 6A even.tif]

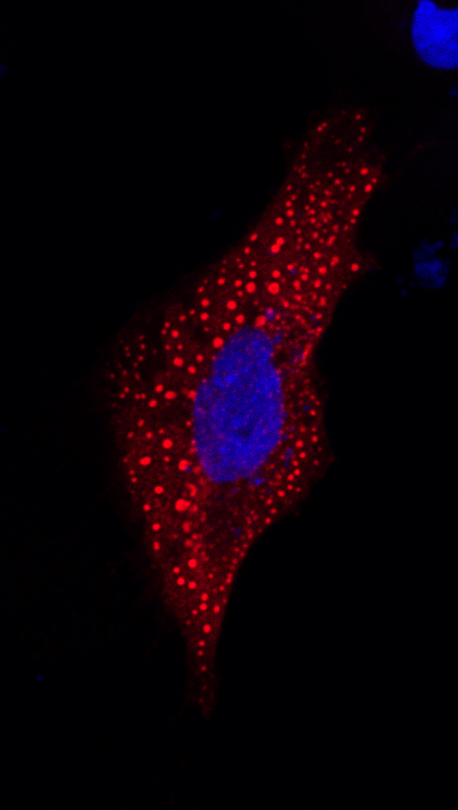

Supplement: Supplementary file 12 — Source data Fig. 6 [file 44318_2024_254_MOESM12_ESM.zip › 116133R_source data Fig 6/Micrographs/Fig 6A punctate.tif]

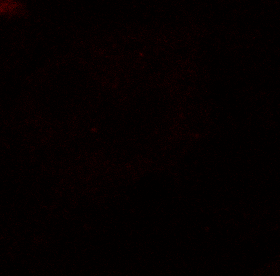

Supplement: Supplementary file 12 — Source data Fig. 6 [file 44318_2024_254_MOESM12_ESM.zip › 116133R_source data Fig 6/Micrographs/FIg 6Da DVL3.tif]

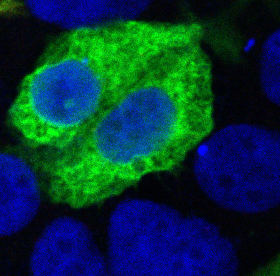

Supplement: Supplementary file 12 — Source data Fig. 6 [file 44318_2024_254_MOESM12_ESM.zip › 116133R_source data Fig 6/Micrographs/Fig 6Da merge.tif]

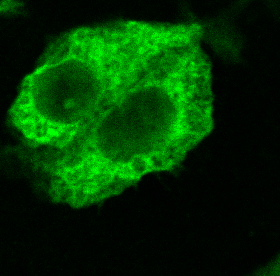

Supplement: Supplementary file 12 — Source data Fig. 6 [file 44318_2024_254_MOESM12_ESM.zip › 116133R_source data Fig 6/Micrographs/Fig 6Da TTLL11.tif]

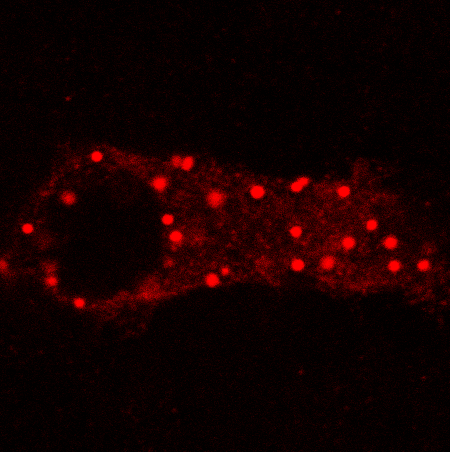

Supplement: Supplementary file 12 — Source data Fig. 6 [file 44318_2024_254_MOESM12_ESM.zip › 116133R_source data Fig 6/Micrographs/Fig 6Db DVL3.tif]

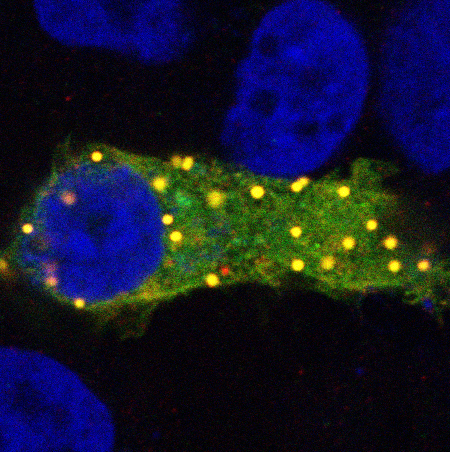

Supplement: Supplementary file 12 — Source data Fig. 6 [file 44318_2024_254_MOESM12_ESM.zip › 116133R_source data Fig 6/Micrographs/Fig 6Db Merge.tif]

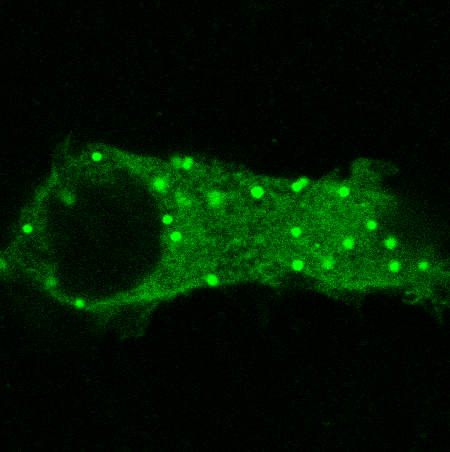

Supplement: Supplementary file 12 — Source data Fig. 6 [file 44318_2024_254_MOESM12_ESM.zip › 116133R_source data Fig 6/Micrographs/Fig 6Db TTLL11.tif]

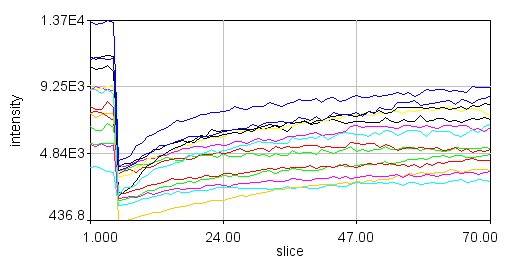

Supplement: Supplementary file 12 — Source data Fig. 6 [file 44318_2024_254_MOESM12_ESM.zip › 116133R_source data Fig 6/Fig 6C FRAP/12E plots/spots/07_12E_spots_combined2.jpg]

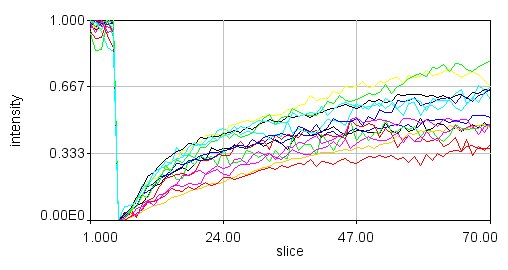

Supplement: Supplementary file 12 — Source data Fig. 6 [file 44318_2024_254_MOESM12_ESM.zip › 116133R_source data Fig 6/Fig 6C FRAP/12E plots/spots/07_12E_spots_combined_normalized2.jpg]

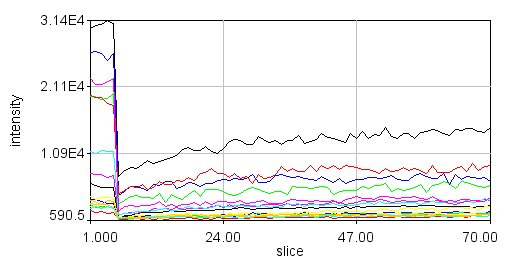

Supplement: Supplementary file 12 — Source data Fig. 6 [file 44318_2024_254_MOESM12_ESM.zip › 116133R_source data Fig 6/Fig 6C FRAP/DCA plots/spots/DCA_spots_combined.jpg]

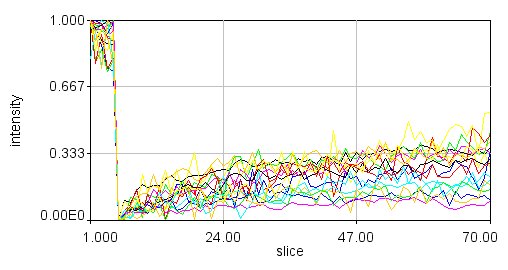

Supplement: Supplementary file 12 — Source data Fig. 6 [file 44318_2024_254_MOESM12_ESM.zip › 116133R_source data Fig 6/Fig 6C FRAP/DCA plots/spots/DCA_spots_combined_normalized.jpg]

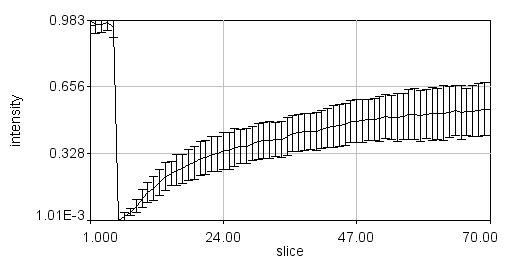

Supplement: Supplementary file 12 — Source data Fig. 6 [file 44318_2024_254_MOESM12_ESM.zip › 116133R_source data Fig 6/Fig 6C FRAP/Results/Spots/12E_spots_average_SD.jpg]

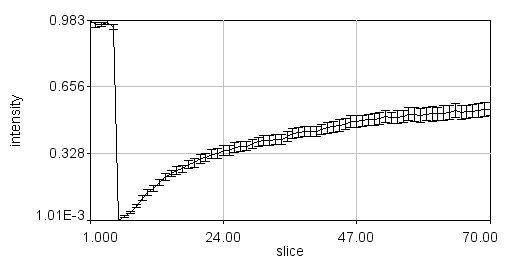

Supplement: Supplementary file 12 — Source data Fig. 6 [file 44318_2024_254_MOESM12_ESM.zip › 116133R_source data Fig 6/Fig 6C FRAP/Results/Spots/12E_spots_average_SE.jpg]

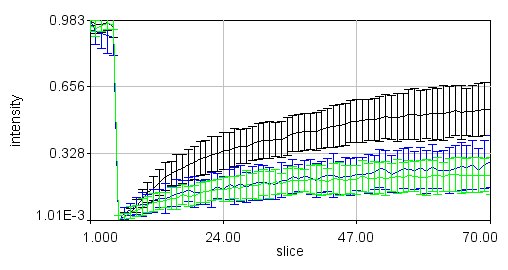

Supplement: Supplementary file 12 — Source data Fig. 6 [file 44318_2024_254_MOESM12_ESM.zip › 116133R_source data Fig 6/Fig 6C FRAP/Results/Spots/all_spots_average_SD.jpg]

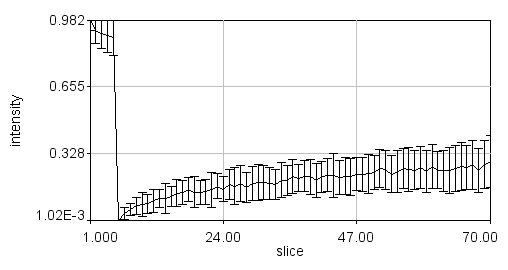

Supplement: Supplementary file 12 — Source data Fig. 6 [file 44318_2024_254_MOESM12_ESM.zip › 116133R_source data Fig 6/Fig 6C FRAP/Results/Spots/DCA_spots_average_SD.jpg]

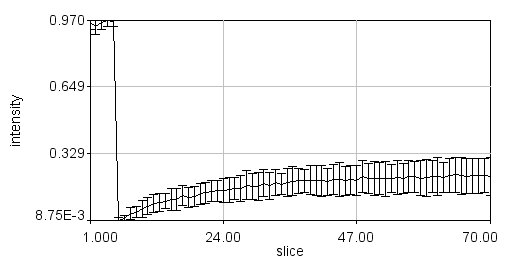

Supplement: Supplementary file 12 — Source data Fig. 6 [file 44318_2024_254_MOESM12_ESM.zip › 116133R_source data Fig 6/Fig 6C FRAP/Results/Spots/WT_spots_average_SD.jpg]

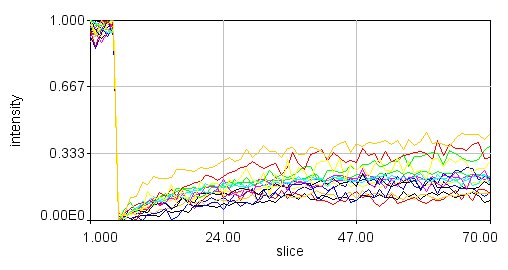

Supplement: Supplementary file 12 — Source data Fig. 6 [file 44318_2024_254_MOESM12_ESM.zip › 116133R_source data Fig 6/Fig 6C FRAP/WT plots/spots/WT_spots_combined_normalized.jpg]

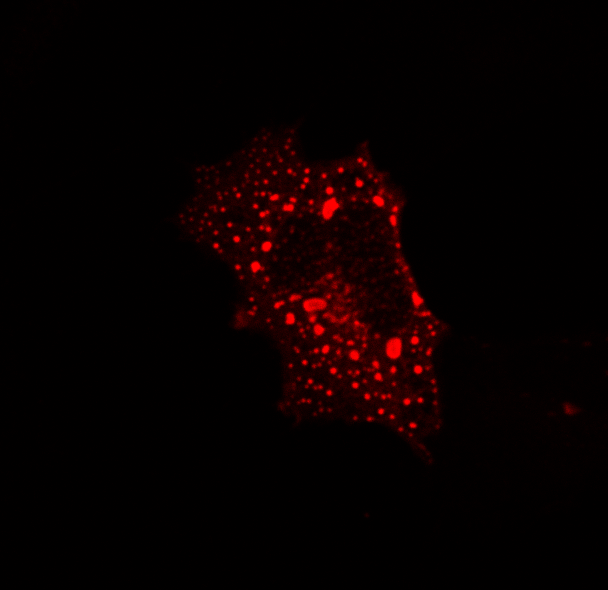

Supplement: Supplementary file 13 — Source data Fig. 7 [file 44318_2024_254_MOESM13_ESM.zip › 116133R_source data Fig 7/Micrographs/Fig 7A CCP1 DVL3 2.tif]

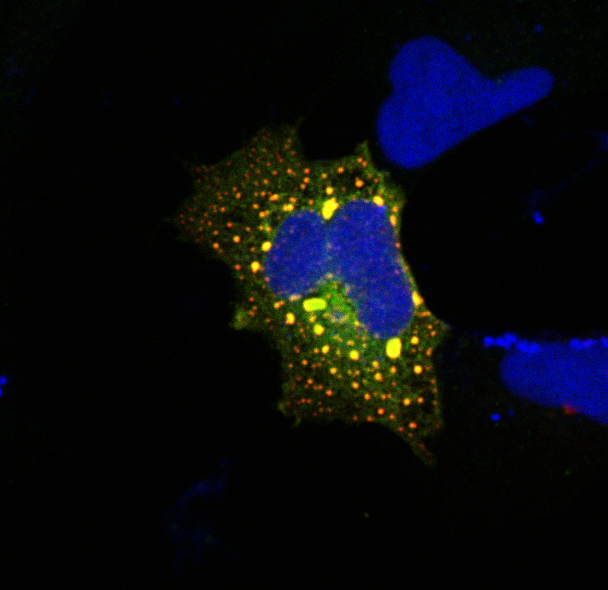

Supplement: Supplementary file 13 — Source data Fig. 7 [file 44318_2024_254_MOESM13_ESM.zip › 116133R_source data Fig 7/Micrographs/Fig 7A CCP1 DVL3 merge.tif]

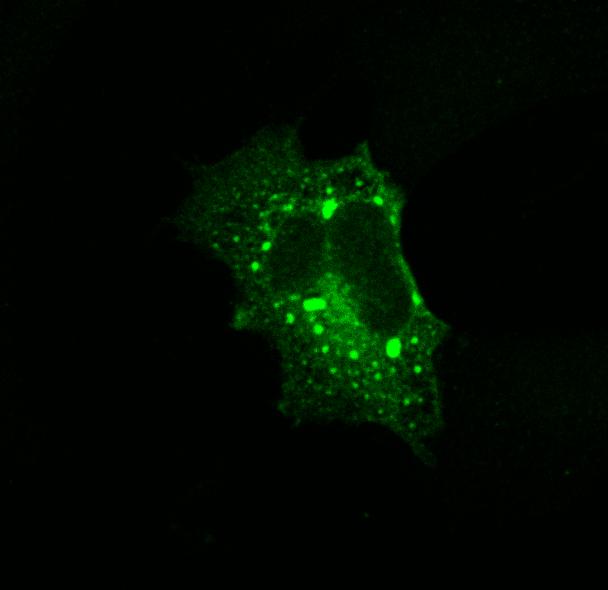

Supplement: Supplementary file 13 — Source data Fig. 7 [file 44318_2024_254_MOESM13_ESM.zip › 116133R_source data Fig 7/Micrographs/Fig 7A CCP1 DVL3.tif]

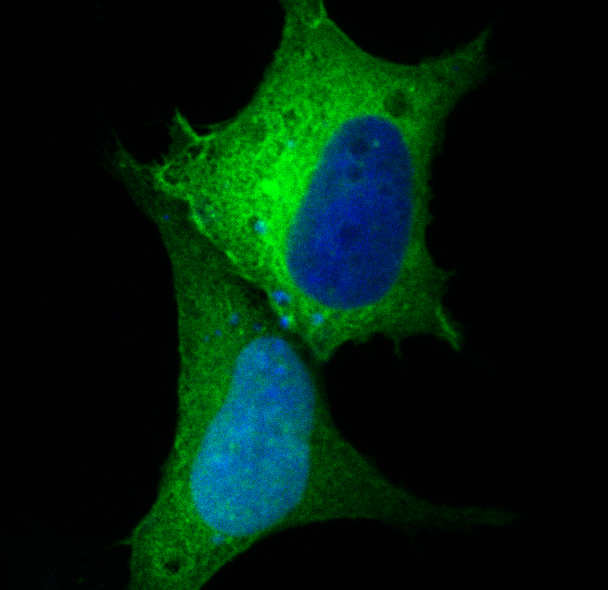

Supplement: Supplementary file 13 — Source data Fig. 7 [file 44318_2024_254_MOESM13_ESM.zip › 116133R_source data Fig 7/Micrographs/FIg 7A CCP1.tif]

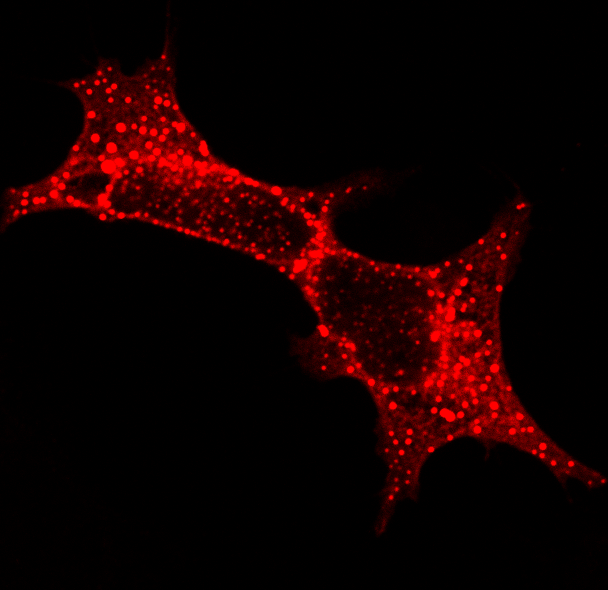

Supplement: Supplementary file 13 — Source data Fig. 7 [file 44318_2024_254_MOESM13_ESM.zip › 116133R_source data Fig 7/Micrographs/Fig 7A CCP5 DVL3 2.tif]

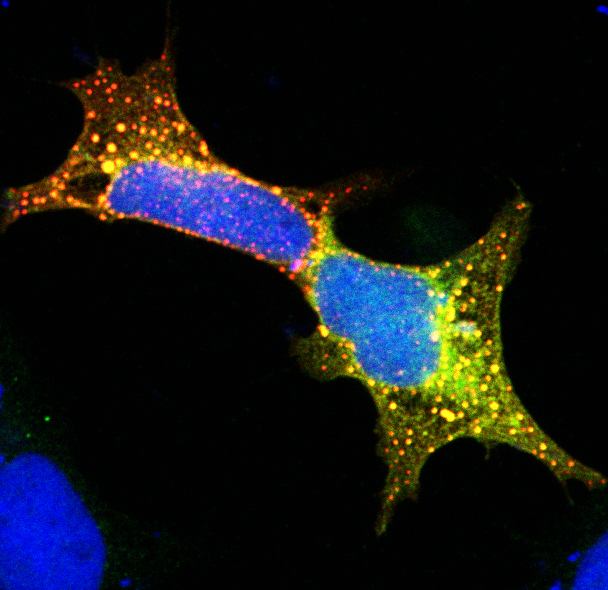

Supplement: Supplementary file 13 — Source data Fig. 7 [file 44318_2024_254_MOESM13_ESM.zip › 116133R_source data Fig 7/Micrographs/Fig 7A CCP5 DVL3 merge.tif]

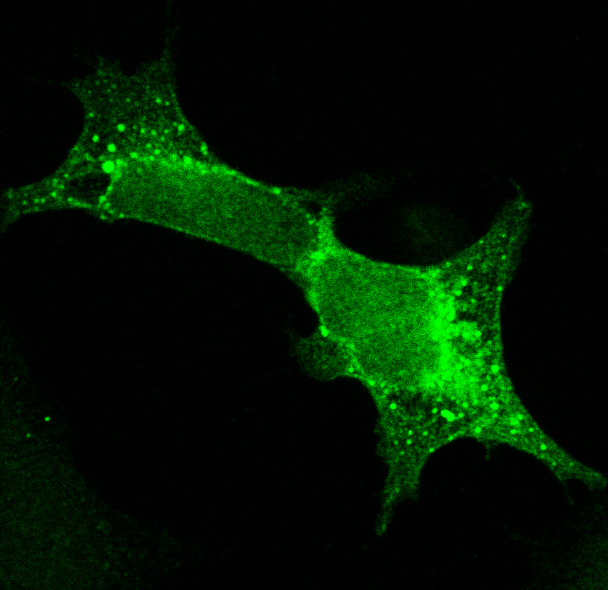

Supplement: Supplementary file 13 — Source data Fig. 7 [file 44318_2024_254_MOESM13_ESM.zip › 116133R_source data Fig 7/Micrographs/Fig 7A CCP5 DVL3.tif]

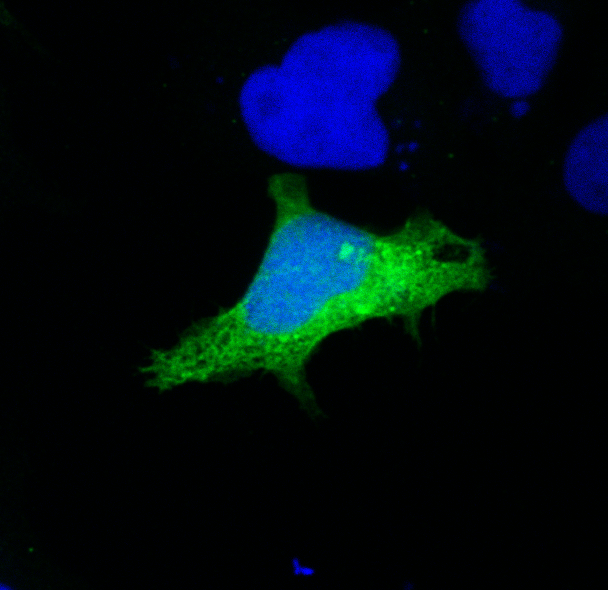

Supplement: Supplementary file 13 — Source data Fig. 7 [file 44318_2024_254_MOESM13_ESM.zip › 116133R_source data Fig 7/Micrographs/Fig 7A CCP5.tif]

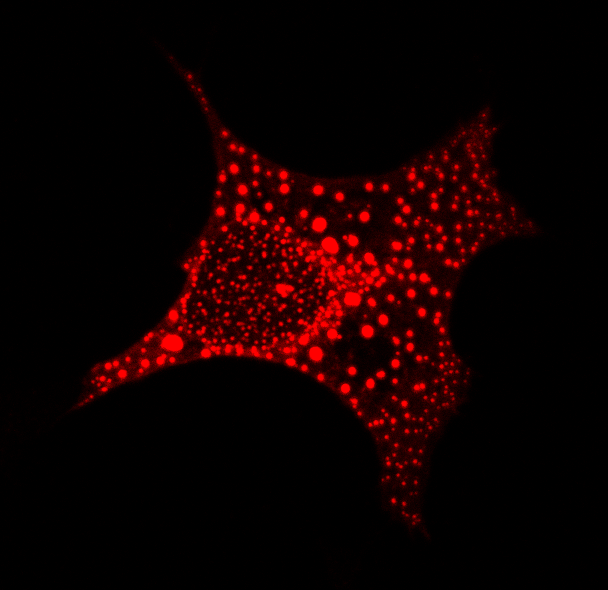

Supplement: Supplementary file 13 — Source data Fig. 7 [file 44318_2024_254_MOESM13_ESM.zip › 116133R_source data Fig 7/Micrographs/Fig 7A CCP6 DVL3 2.tif]

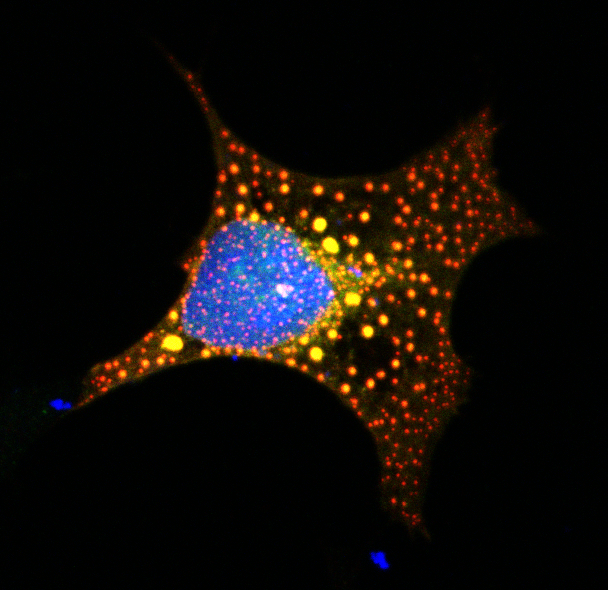

Supplement: Supplementary file 13 — Source data Fig. 7 [file 44318_2024_254_MOESM13_ESM.zip › 116133R_source data Fig 7/Micrographs/Fig 7A CCP6 DVL3 merge.tif]

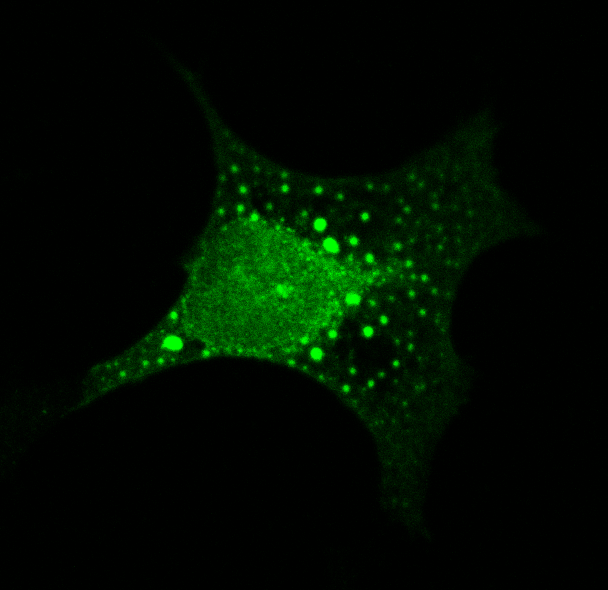

Supplement: Supplementary file 13 — Source data Fig. 7 [file 44318_2024_254_MOESM13_ESM.zip › 116133R_source data Fig 7/Micrographs/Fig 7A CCP6 DVL3.tif]

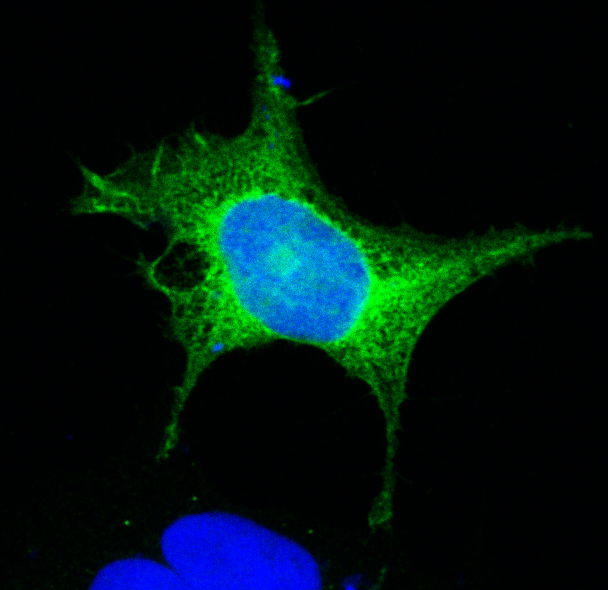

Supplement: Supplementary file 13 — Source data Fig. 7 [file 44318_2024_254_MOESM13_ESM.zip › 116133R_source data Fig 7/Micrographs/Fig 7A CCP6.tif]

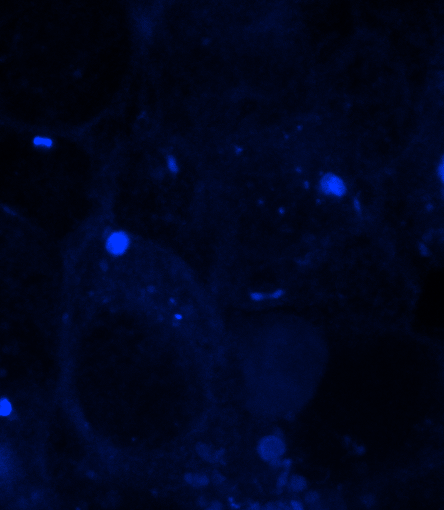

Supplement: Supplementary file 14 — Source data Fig. 8 [file 44318_2024_254_MOESM14_ESM.zip › 116133R_source data Fig 8/Micrographs/Fig 8B Centrosome/ECFP-centrosome + DOX.png]

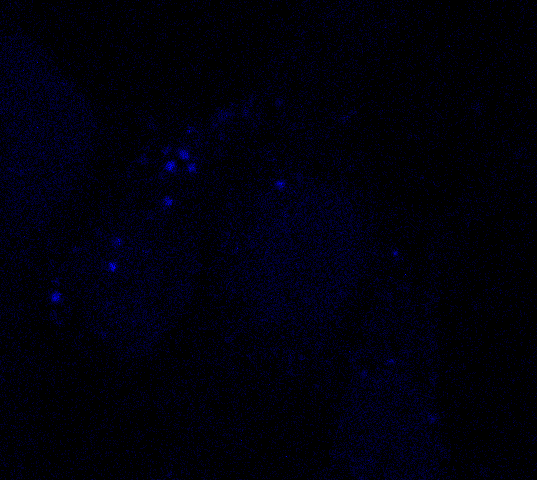

Supplement: Supplementary file 14 — Source data Fig. 8 [file 44318_2024_254_MOESM14_ESM.zip › 116133R_source data Fig 8/Micrographs/Fig 8B Centrosome/ECFP-centrosome no DOX.png]

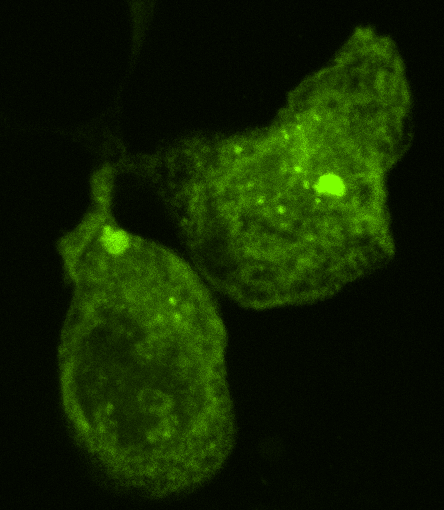

Supplement: Supplementary file 14 — Source data Fig. 8 [file 44318_2024_254_MOESM14_ESM.zip › 116133R_source data Fig 8/Micrographs/Fig 8B Centrosome/EYFP-centrosome + DOX.png]

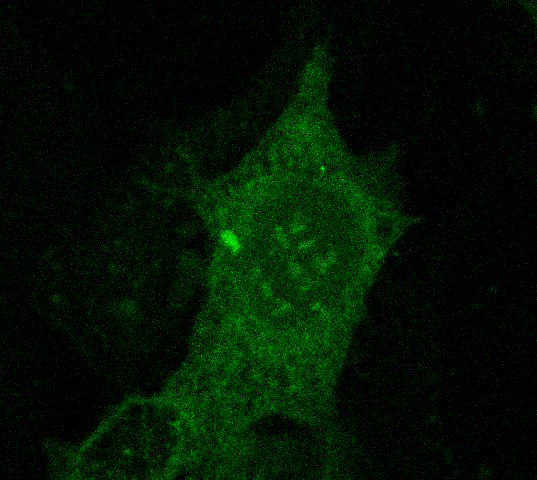

Supplement: Supplementary file 14 — Source data Fig. 8 [file 44318_2024_254_MOESM14_ESM.zip › 116133R_source data Fig 8/Micrographs/Fig 8B Centrosome/EYFP-centrosome no DOX.png]

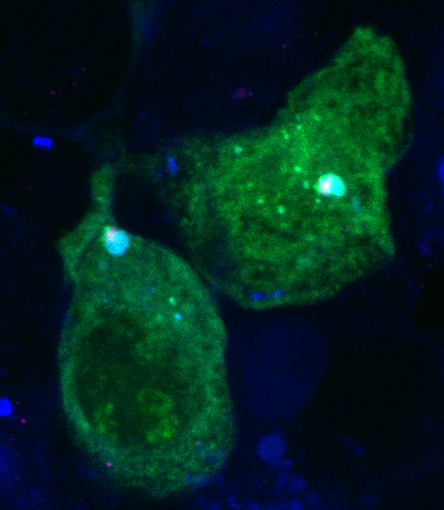

Supplement: Supplementary file 14 — Source data Fig. 8 [file 44318_2024_254_MOESM14_ESM.zip › 116133R_source data Fig 8/Micrographs/Fig 8B Centrosome/merge-centrosome + DOX.png]

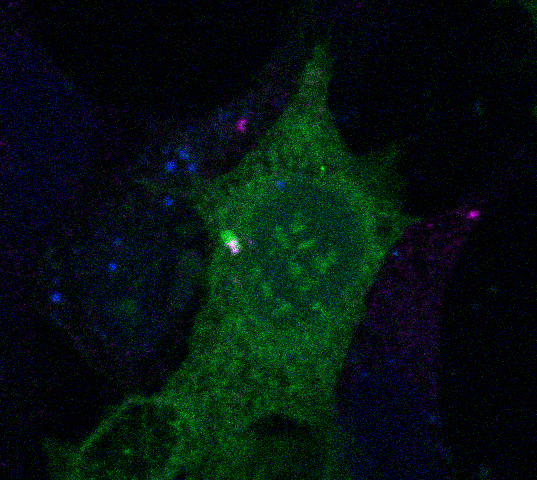

Supplement: Supplementary file 14 — Source data Fig. 8 [file 44318_2024_254_MOESM14_ESM.zip › 116133R_source data Fig 8/Micrographs/Fig 8B Centrosome/merge-centrosome no DOX.png]

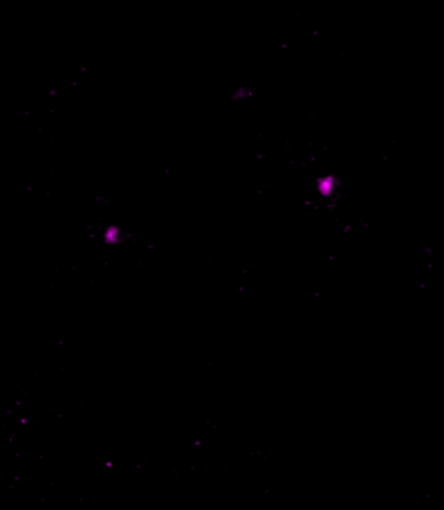

Supplement: Supplementary file 14 — Source data Fig. 8 [file 44318_2024_254_MOESM14_ESM.zip › 116133R_source data Fig 8/Micrographs/Fig 8B Centrosome/Pericentrin-centrosome + DOX.png]

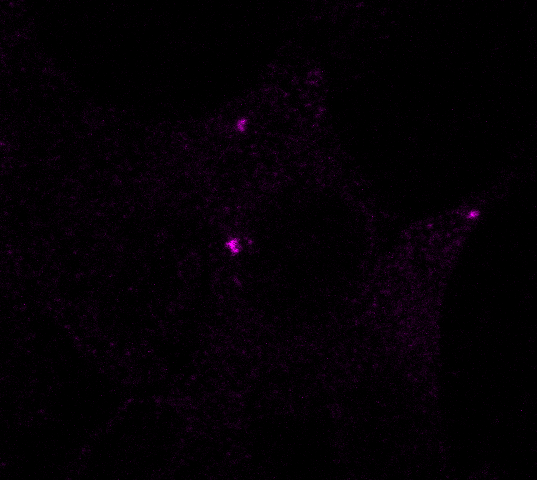

Supplement: Supplementary file 14 — Source data Fig. 8 [file 44318_2024_254_MOESM14_ESM.zip › 116133R_source data Fig 8/Micrographs/Fig 8B Centrosome/Pericentrin-centrosome no DOX.png]

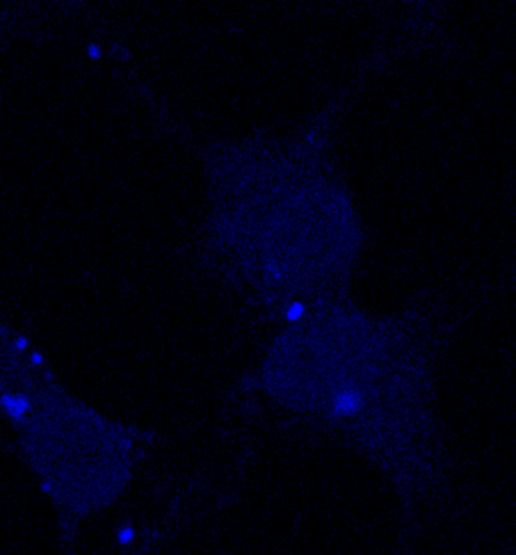

Supplement: Supplementary file 14 — Source data Fig. 8 [file 44318_2024_254_MOESM14_ESM.zip › 116133R_source data Fig 8/Micrographs/Fig 8B Midbody/ECFP-midbody + DOX.png]

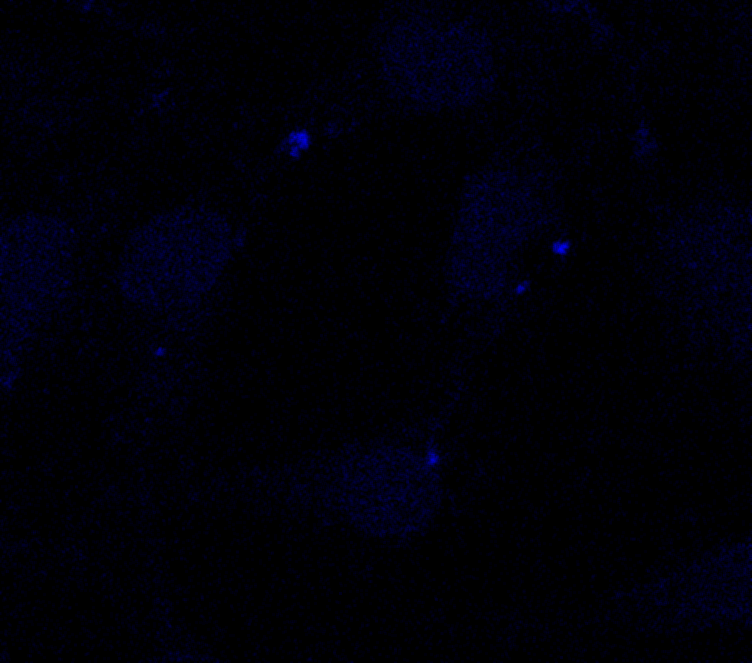

Supplement: Supplementary file 14 — Source data Fig. 8 [file 44318_2024_254_MOESM14_ESM.zip › 116133R_source data Fig 8/Micrographs/Fig 8B Midbody/ECFP-midbody no DOX.png]

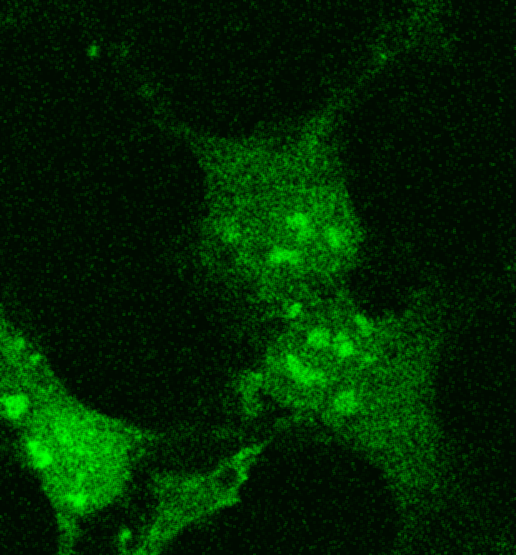

Supplement: Supplementary file 14 — Source data Fig. 8 [file 44318_2024_254_MOESM14_ESM.zip › 116133R_source data Fig 8/Micrographs/Fig 8B Midbody/EYFP-midbody + DOX.png]

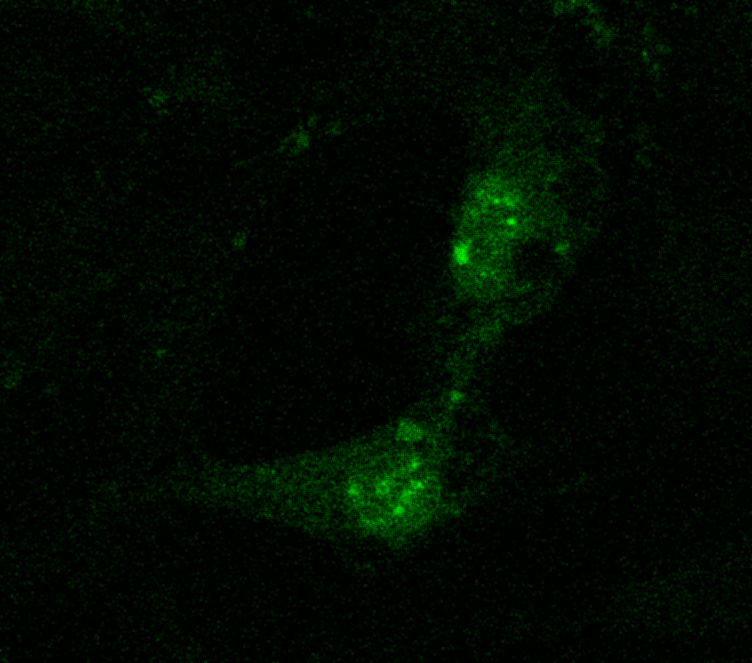

Supplement: Supplementary file 14 — Source data Fig. 8 [file 44318_2024_254_MOESM14_ESM.zip › 116133R_source data Fig 8/Micrographs/Fig 8B Midbody/EYFP-midbody no DOX.png]

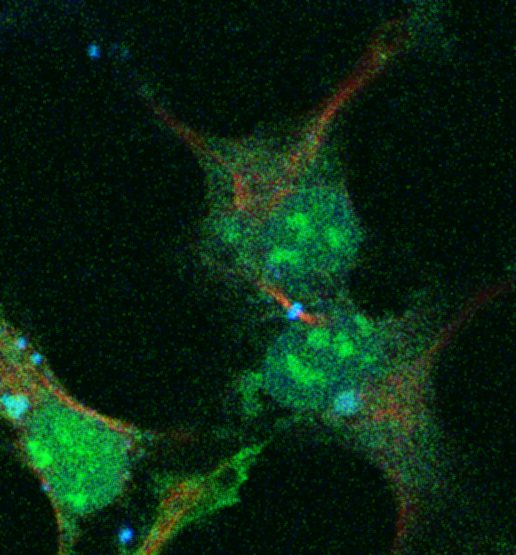

Supplement: Supplementary file 14 — Source data Fig. 8 [file 44318_2024_254_MOESM14_ESM.zip › 116133R_source data Fig 8/Micrographs/Fig 8B Midbody/merge-midbody + DOX.png]

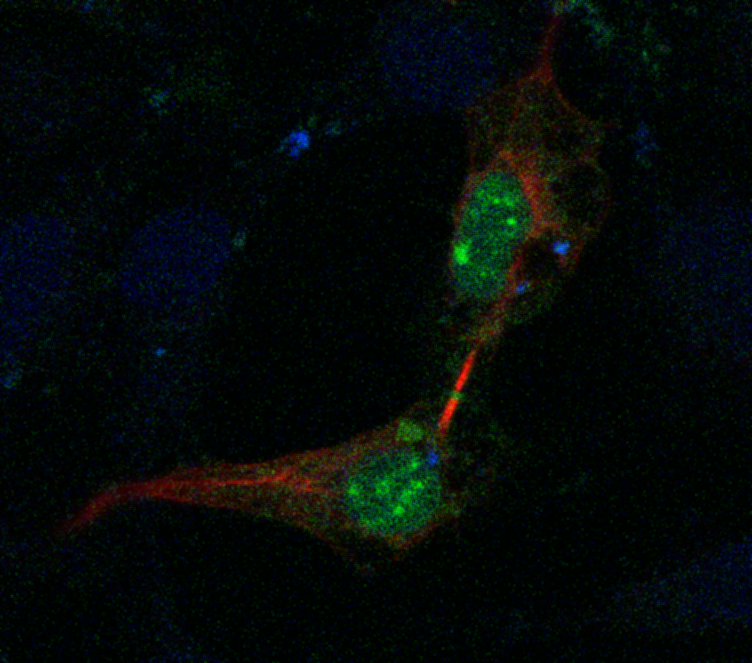

Supplement: Supplementary file 14 — Source data Fig. 8 [file 44318_2024_254_MOESM14_ESM.zip › 116133R_source data Fig 8/Micrographs/Fig 8B Midbody/merge-midbody no DOX.png]

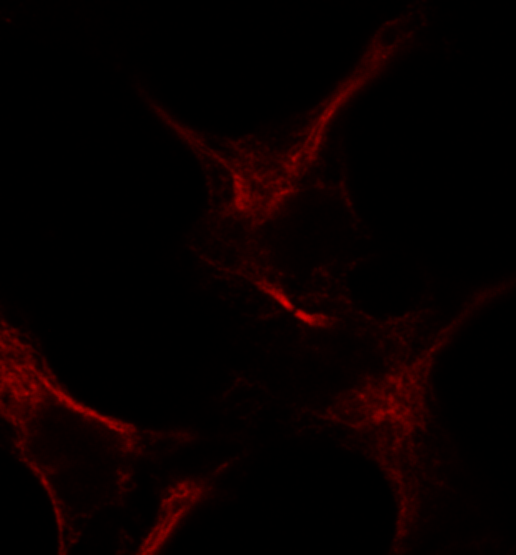

Supplement: Supplementary file 14 — Source data Fig. 8 [file 44318_2024_254_MOESM14_ESM.zip › 116133R_source data Fig 8/Micrographs/Fig 8B Midbody/PolyE-midbody + DOX.png]

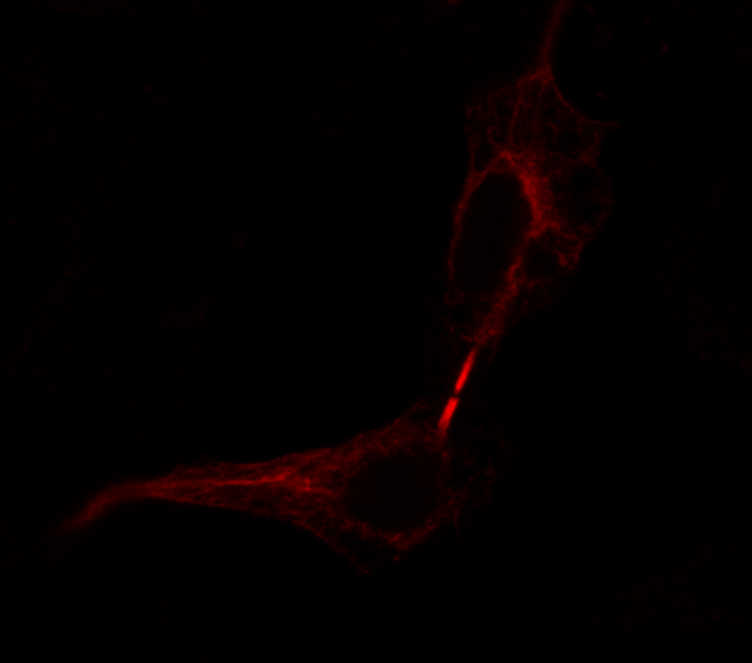

Supplement: Supplementary file 14 — Source data Fig. 8 [file 44318_2024_254_MOESM14_ESM.zip › 116133R_source data Fig 8/Micrographs/Fig 8B Midbody/PolyE-midbody no DOX.png]

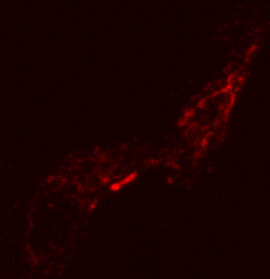

Supplement: Supplementary file 14 — Source data Fig. 8 [file 44318_2024_254_MOESM14_ESM.zip › 116133R_source data Fig 8/Micrographs/Fig 8B Primary cilium/AcTubulin-primary cilium + DOX.png]

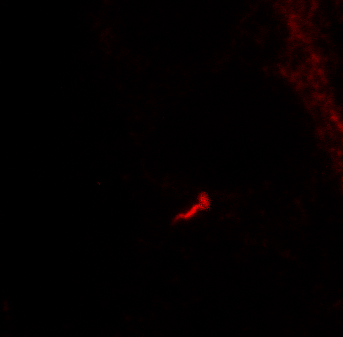

Supplement: Supplementary file 14 — Source data Fig. 8 [file 44318_2024_254_MOESM14_ESM.zip › 116133R_source data Fig 8/Micrographs/Fig 8B Primary cilium/AcTubulin-primary cilium no DOX.png]

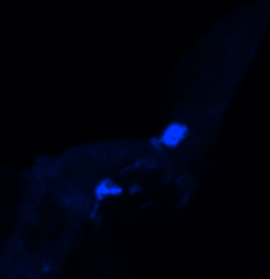

Supplement: Supplementary file 14 — Source data Fig. 8 [file 44318_2024_254_MOESM14_ESM.zip › 116133R_source data Fig 8/Micrographs/Fig 8B Primary cilium/ECFP-primary cilium + DOX.png]

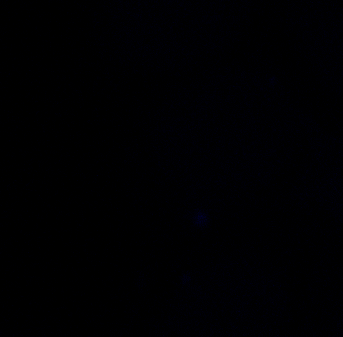

Supplement: Supplementary file 14 — Source data Fig. 8 [file 44318_2024_254_MOESM14_ESM.zip › 116133R_source data Fig 8/Micrographs/Fig 8B Primary cilium/ECFP-primary cilium no DOX.png]

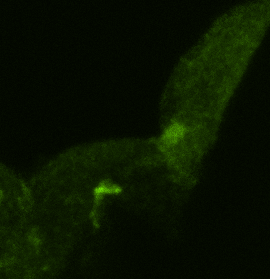

Supplement: Supplementary file 14 — Source data Fig. 8 [file 44318_2024_254_MOESM14_ESM.zip › 116133R_source data Fig 8/Micrographs/Fig 8B Primary cilium/EYFP-primary cilium + DOX.png]

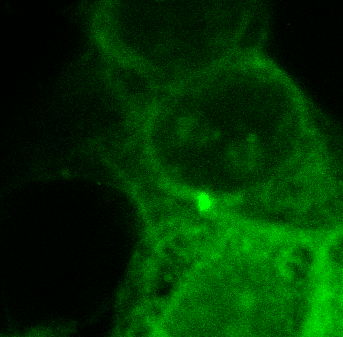

Supplement: Supplementary file 14 — Source data Fig. 8 [file 44318_2024_254_MOESM14_ESM.zip › 116133R_source data Fig 8/Micrographs/Fig 8B Primary cilium/EYFP-primary cilium no DOX.png]

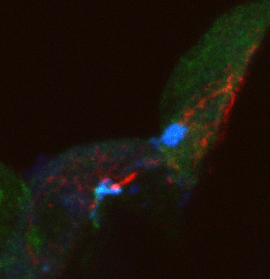

Supplement: Supplementary file 14 — Source data Fig. 8 [file 44318_2024_254_MOESM14_ESM.zip › 116133R_source data Fig 8/Micrographs/Fig 8B Primary cilium/merge-primary cilium + DOX.png]

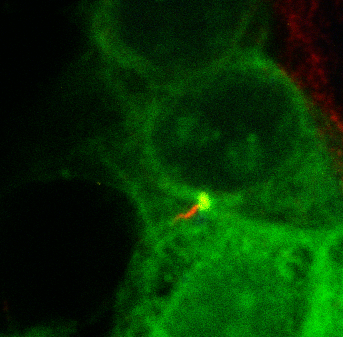

Supplement: Supplementary file 14 — Source data Fig. 8 [file 44318_2024_254_MOESM14_ESM.zip › 116133R_source data Fig 8/Micrographs/Fig 8B Primary cilium/merge-primary cilium no DOX.png]

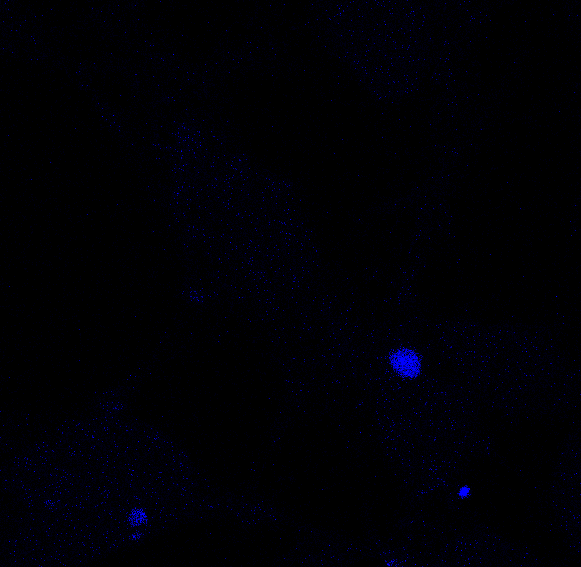

Supplement: Supplementary file 14 — Source data Fig. 8 [file 44318_2024_254_MOESM14_ESM.zip › 116133R_source data Fig 8/Micrographs/Fig 8C/ECFP + DOX.png]

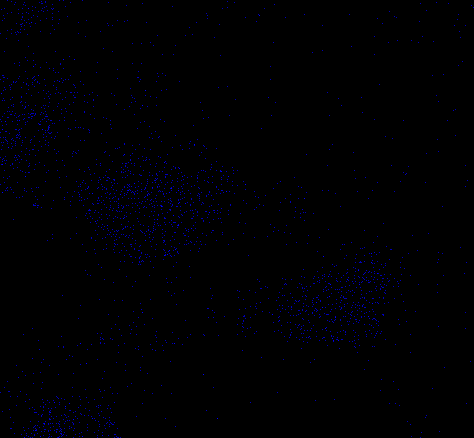

Supplement: Supplementary file 14 — Source data Fig. 8 [file 44318_2024_254_MOESM14_ESM.zip › 116133R_source data Fig 8/Micrographs/Fig 8C/ECFP no DOX.png]

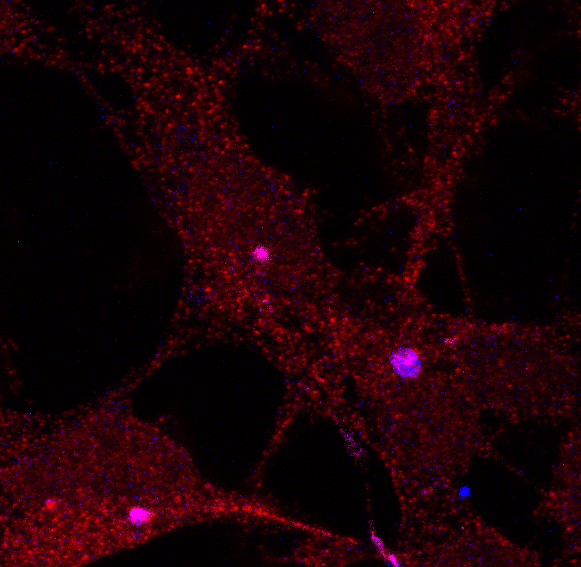

Supplement: Supplementary file 14 — Source data Fig. 8 [file 44318_2024_254_MOESM14_ESM.zip › 116133R_source data Fig 8/Micrographs/Fig 8C/Merge + DOX.png]

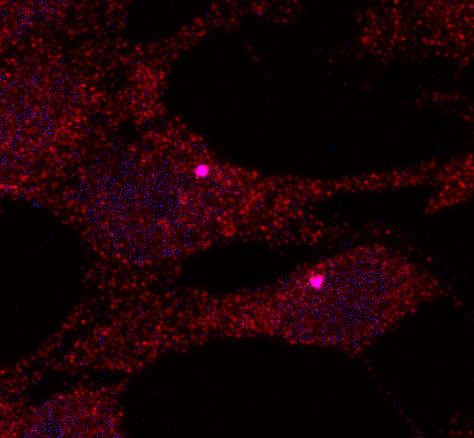

Supplement: Supplementary file 14 — Source data Fig. 8 [file 44318_2024_254_MOESM14_ESM.zip › 116133R_source data Fig 8/Micrographs/Fig 8C/merge no DOX.png]

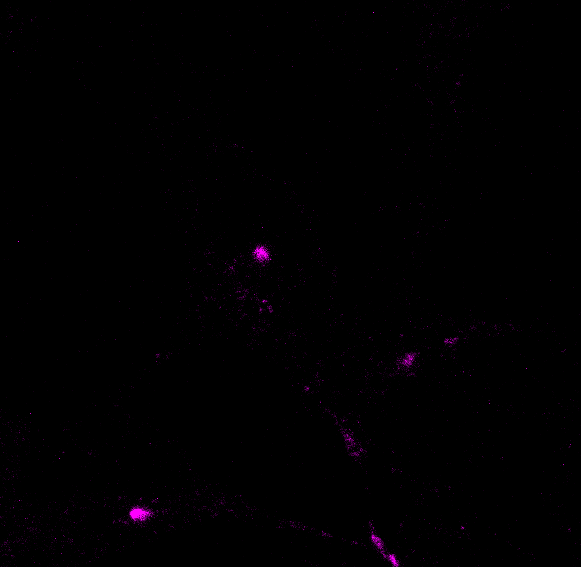

Supplement: Supplementary file 14 — Source data Fig. 8 [file 44318_2024_254_MOESM14_ESM.zip › 116133R_source data Fig 8/Micrographs/Fig 8C/Pericentrin + DOX.png]

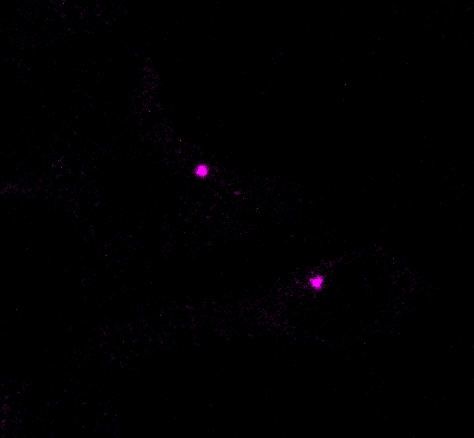

Supplement: Supplementary file 14 — Source data Fig. 8 [file 44318_2024_254_MOESM14_ESM.zip › 116133R_source data Fig 8/Micrographs/Fig 8C/Pericentrin no DOX.png]

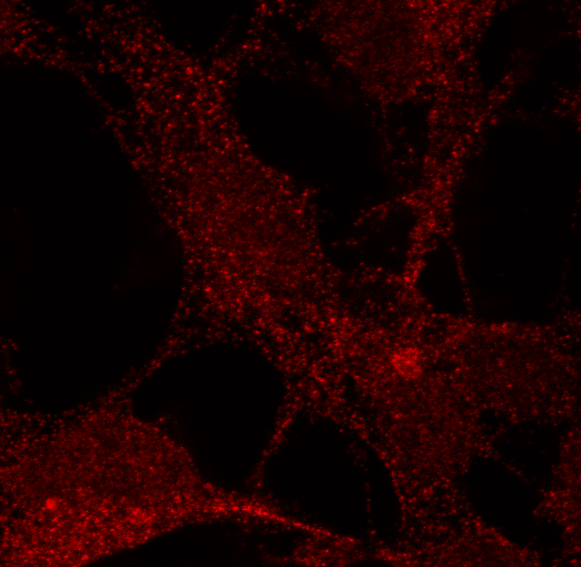

Supplement: Supplementary file 14 — Source data Fig. 8 [file 44318_2024_254_MOESM14_ESM.zip › 116133R_source data Fig 8/Micrographs/Fig 8C/PolyE + DOX.png]

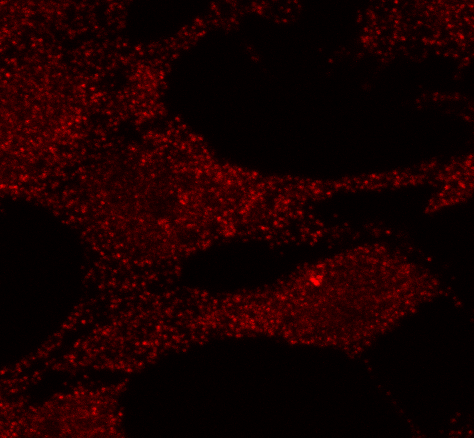

Supplement: Supplementary file 14 — Source data Fig. 8 [file 44318_2024_254_MOESM14_ESM.zip › 116133R_source data Fig 8/Micrographs/Fig 8C/PolyE no DOX.png]

## Slide 1
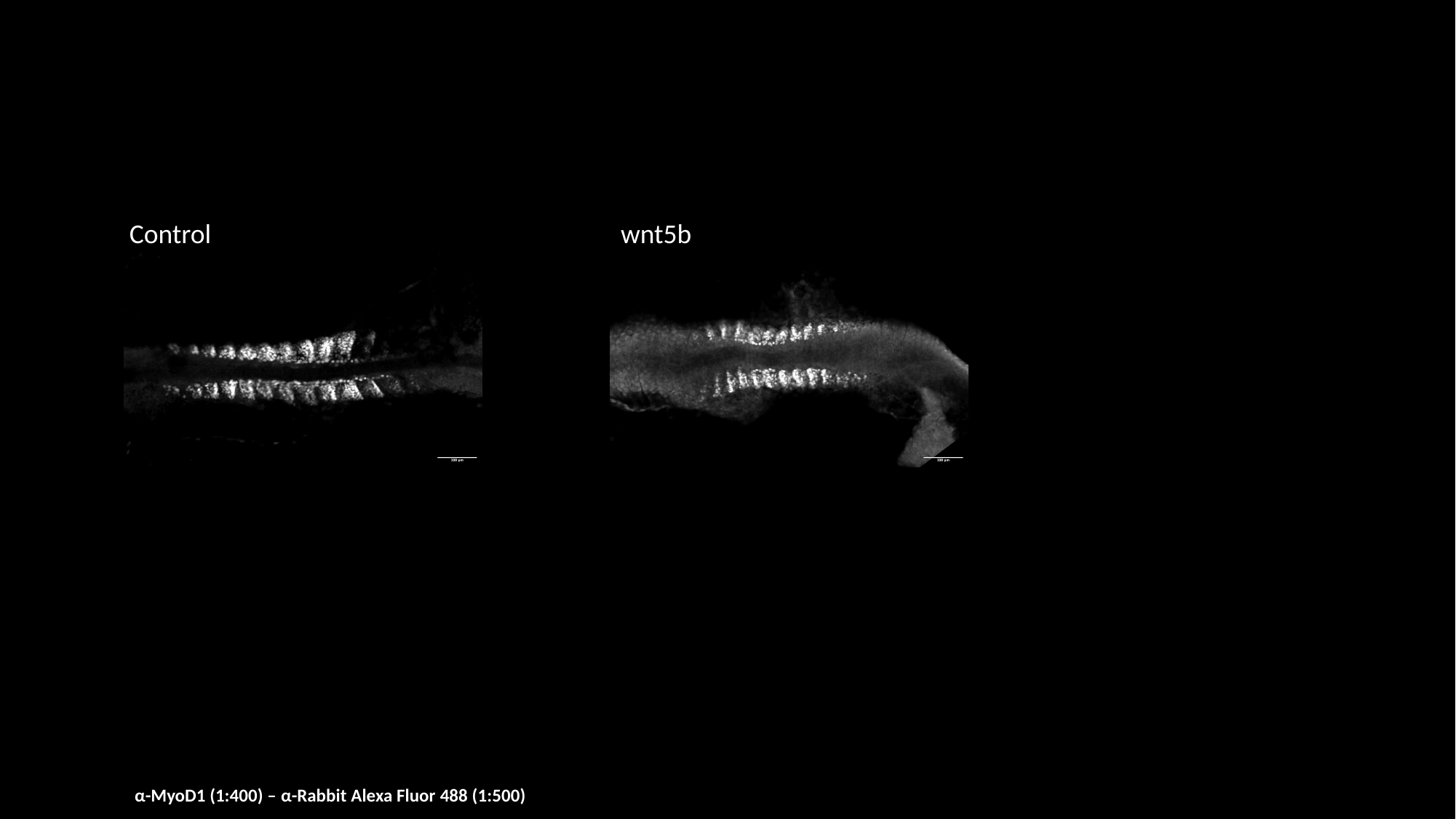

Control
wnt5b
α-MyoD1 (1:400) – α-Rabbit Alexa Fluor 488 (1:500)

Supplement: Supplementary file 15 — EV and Appendix Source Data [file 44318_2024_254_MOESM15_ESM.zip › 116133R_Source Data for Expanded View and Appendix/116133R_source data EV Files/116133R_source data Fig EV5/Source mircographs Fig. EV5H.pptx]

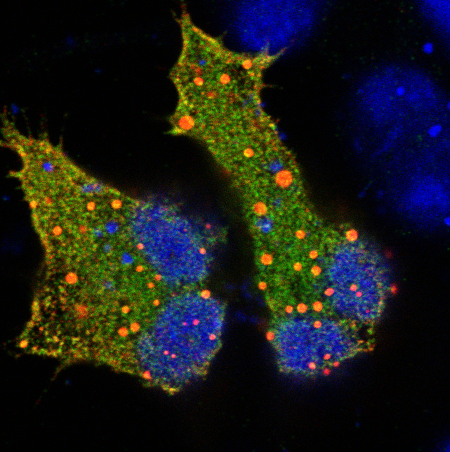

Supplement: Supplementary file 15 — EV and Appendix Source Data [file 44318_2024_254_MOESM15_ESM.zip › 116133R_Source Data for Expanded View and Appendix/116133R_source data Appendix Files/116133R_source data Appendix Fig S3/Micrographs/Fig AS3 D DVL1 TTLL11 merge.tif]

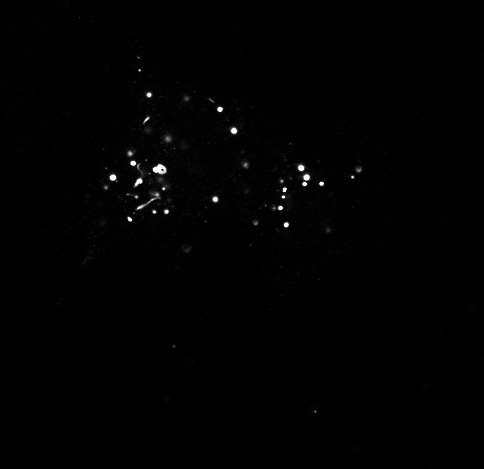

Supplement: Supplementary file 15 — EV and Appendix Source Data [file 44318_2024_254_MOESM15_ESM.zip › 116133R_Source Data for Expanded View and Appendix/116133R_source data Appendix Files/116133R_source data Appendix Fig S3/Micrographs/Fig AS3 D DVL2 TTLL11 merge.tif]
