# Supplementary material for: Carboxy-terminal polyglutamylation regulates signaling and phase separation of the Dishevelled protein
Source: EMBO J. 2024 Sep 30;43(22):15. doi: 10.1038/s44318-024-00254-7 (PMC11574253; doi:10.1038/s44318-024-00254-7)
Supplement: Supplementary file 16 — Expanded View Figures [file 44318_2024_254_MOESM16_ESM.pdf]

Expanded View Figures

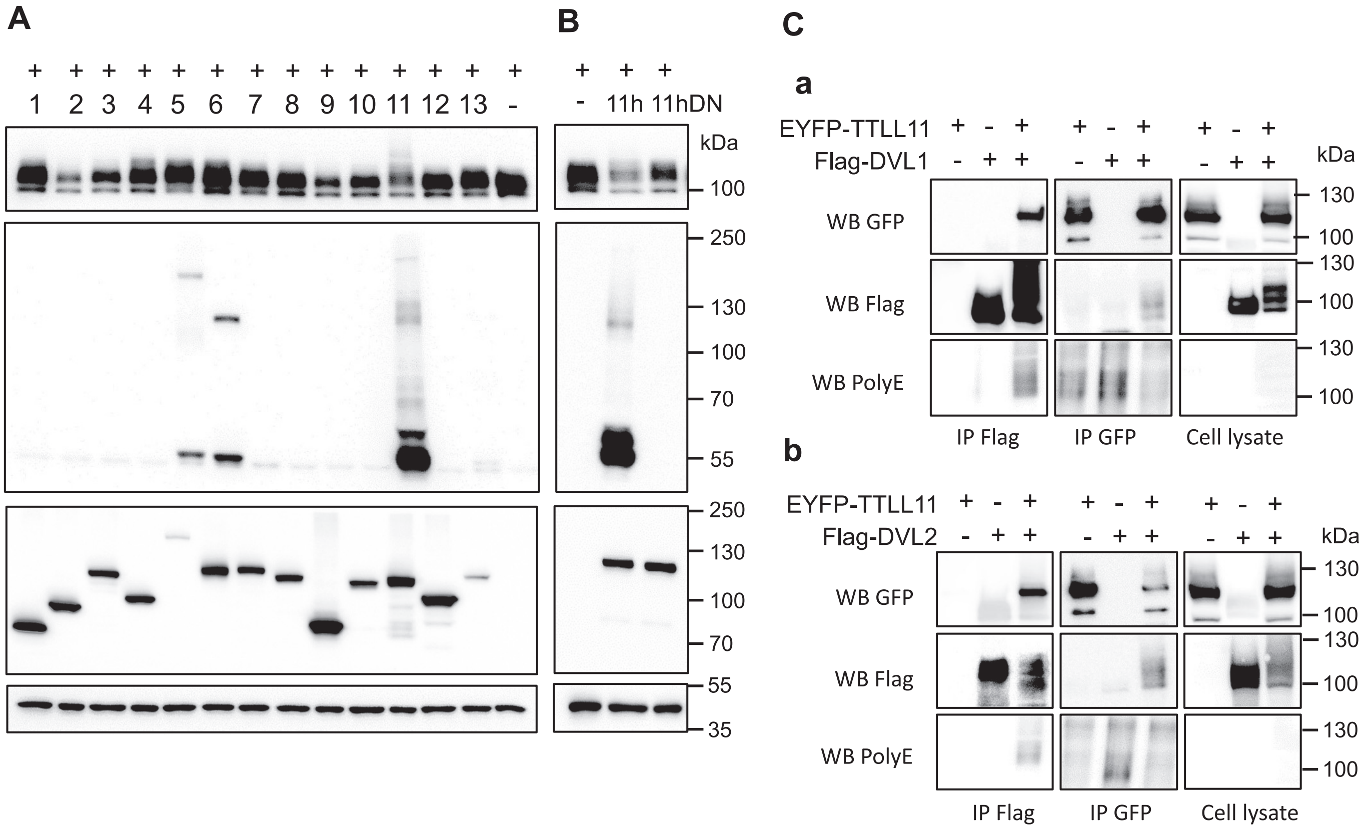

Figure EV1. TTLL11 binds and polyglutamylates DVL1 and DVL2.

(**A**, **B**) HEK293T cells were transfected with constructs encoding murine YFP tagged TTLL1 – TTLL13 (**A**) or with human TTLL11 and its inactive variant TTLL11 (E531G; DN) (**B**) together with Flag-DVL2. The samples were subjected to WB analysis using polyglutamylation specific antibody—PolyE. Note the appearance of polyE-positive bands of DVL2 size in conditions where TTLL11 and DVL2 were co-expressed. (**C**) Co-immunoprecipitation of Flag-DVL1 (**a**) or Flag-DVL2 (**b**) with TTLL11 overexpressed in HEK293T cells. TTLL11 was co-immunoprecipitated with both DVL1 and DVL2 and both DVLs were polyglutamylated in the pulldown when co-expressed with TTLL11 (IP Flag, WB PolyE). Source data are available online for this figure.

 © The Author(s)

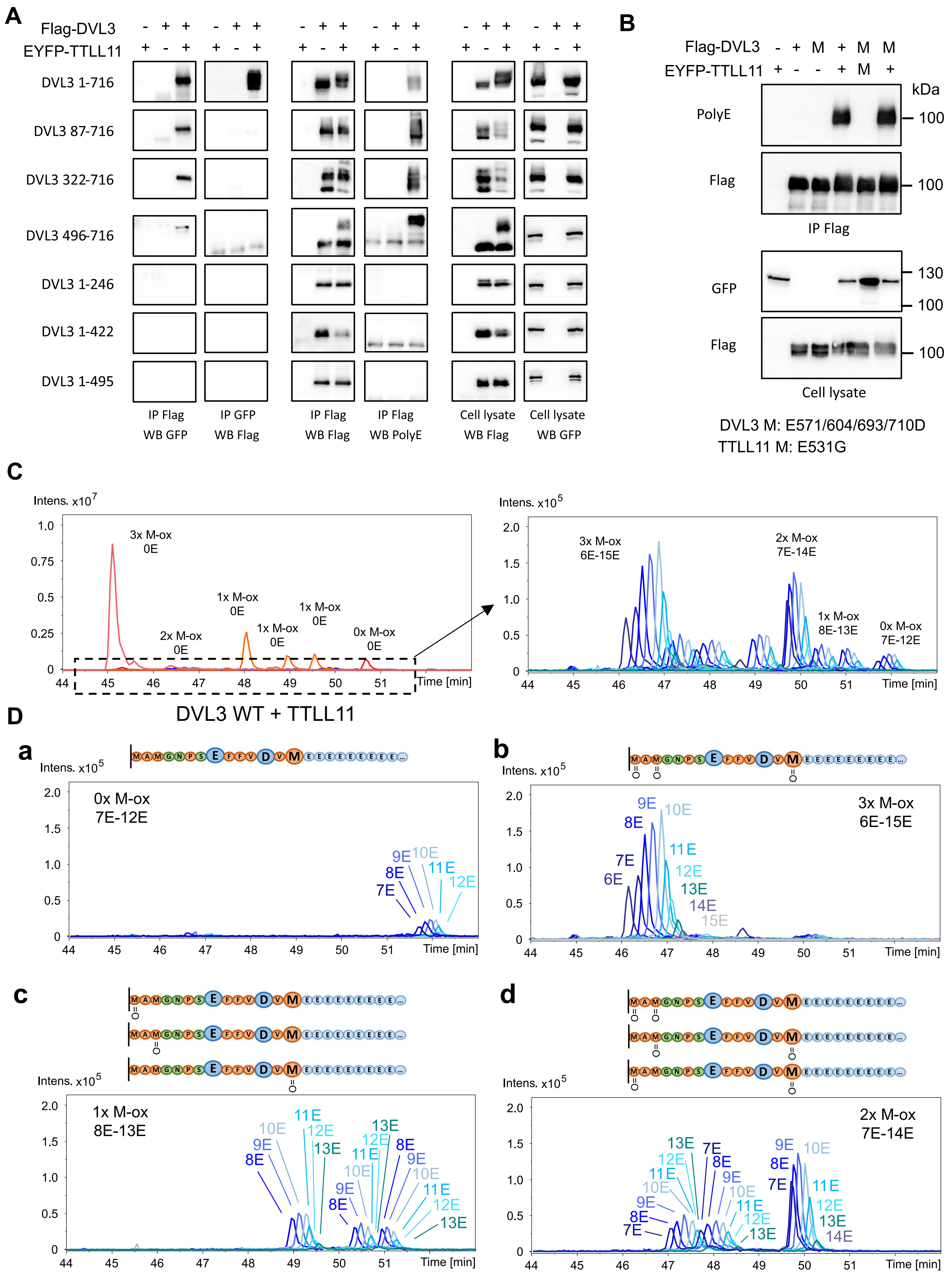
A
Flag-DVL3
EYFP-TTLL11
DVL3 1-716
DVL3 87-716
DVL3 322-716
DVL3 496-716
DVL3 1-246
DVL3 1-422
DVL3 1-495
IP Flag WB GFP
IP GFP WB Flag
IP Flag WB Flag
IP Flag WB PolyE
Cell lysate WB Flag
Cell lysate WB GFP
B
Flag-DVL3
EYFP-TTLL11
kDa
PolyE
Flag
IP Flag
GFP
Cell lysate
DVL3 M: E571/604/693/710D
TTLL11 M: E531G
C
Intens. x10^7
3x M-ox 0E
2x M-ox 0E
1x M-ox 0E
0x M-ox 0E
Time [min]
Intens. x10^5
3x M-ox 6E-15E
2x M-ox 7E-14E
1x M-ox 8E-13E
0x M-ox 7E-12E
D
DVL3 WT + TTLL11
a
0x M-ox 7E-12E
b
3x M-ox 6E-15E
c
1x M-ox 8E-13E
d
2x M-ox 7E-14E

Figure EV2. Modification site of DVL3 polyglutamylation.

(**A**) Domain mapping of DVL3 polyglutamylation and search for TTLL11 interaction domain on DVL3. DVL3 and its truncated mutants were co-expressed with TTLL11 in HEK293T cells and DVL3 was subsequently immunoprecipitated. Only the DVL3 mutants containing C-terminal part were able to pull down TTLL11. Samples were also stained by modification-specific antibody PolyE, which detected polyglutamylation in all DVL3 mutants containing its C-terminus. (**B**) Polyglutamylation of DVL3 E571D/E604D/E693D/E710D is comparable to DVL3 wt. DVL3 variants were overexpressed with TTLL11 or with its inactive mutant TTLL11 E531G in HEK293 cells and immunoprecipitated via Flag tag and analyzed by WB as indicated (**C**, **D**) EIC chromatogram shows peaks corresponding to the very last C-terminal peptide of DVL3 formed after tryptic cleavage and its polyglutamylated variants, that are highlighted in a separate window. The data are from the same experiment as Fig. EV3A. (**C**) EIC shows peaks corresponding to polyglutamylated peptides shown schematically above each chromatogram. (**D**) chromatograms for non- (**Da**), mono- (**Dc**), di- (**Dd**) and tri-oxidized (**Db**) peptides are presented separately. Source data are available online for this figure.

© The Author(s)

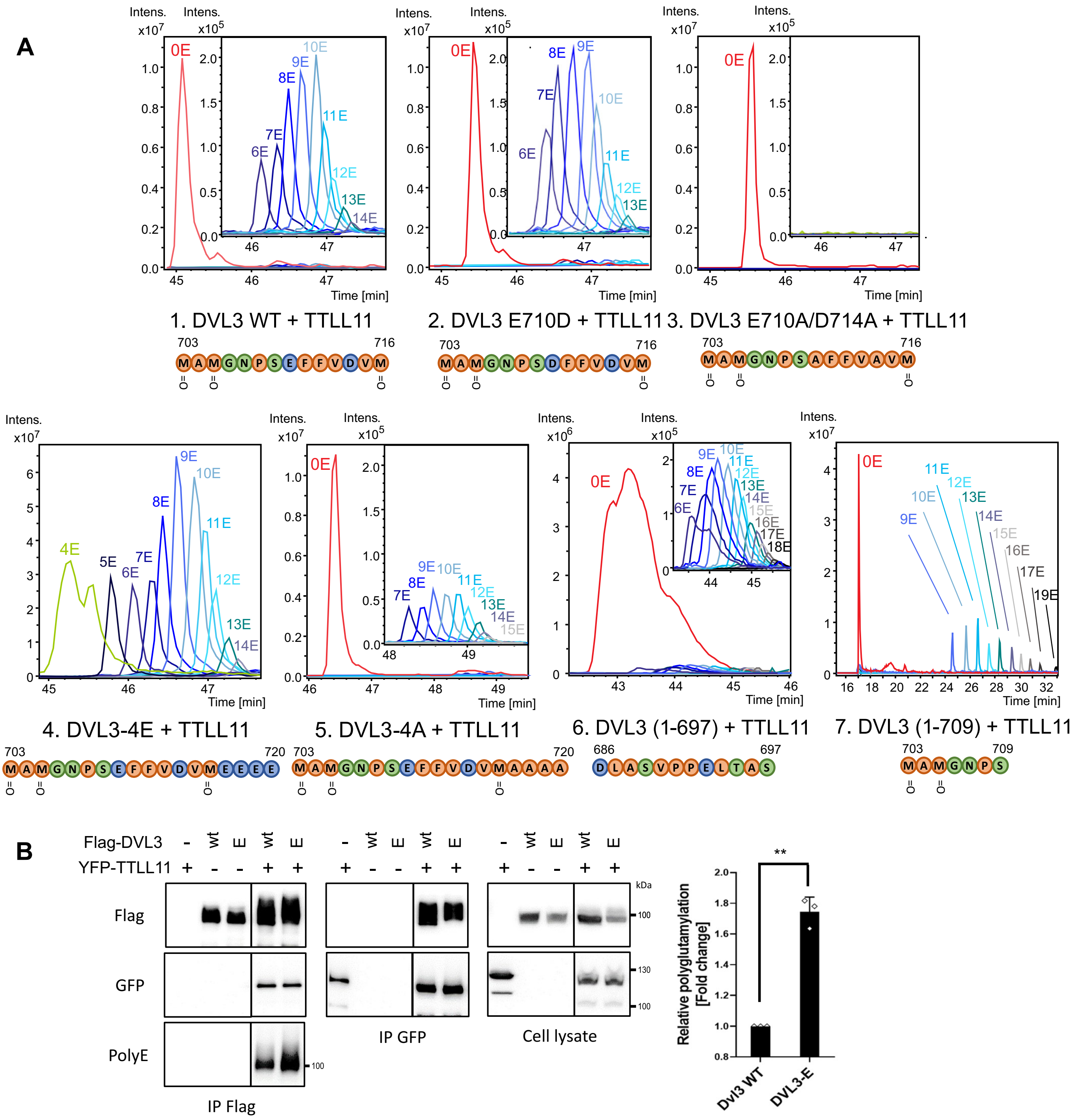

Figure EV3. C-terminal polyglutamylation.

(**A**) EIC showing polyglutamylation of DVL3 C-terminus by TTLL11 in WT protein and mutated variants from Fig. 3A. Lower intensity peaks for polyglutamylated peptides are highlighted in separate windows. Results are shown for fully M-ox peptides. (**B**) Polyglutamylation of Flag-DVL3 C-terminus after addition of 1E residue at the C-terminal M716. Relative polyglutamylation was derived from PolyE band intensity, normalized to total protein (Flag) signal, and is shown as a fold change compared to Dvl3 WT polyglutamylation in three biological replicates. Graph represents mean with SD. Statistical significance was assessed using one-sample t test with theoretical mean = 1; $^{**}P = 0.0055$. Source data are available online for this figure.

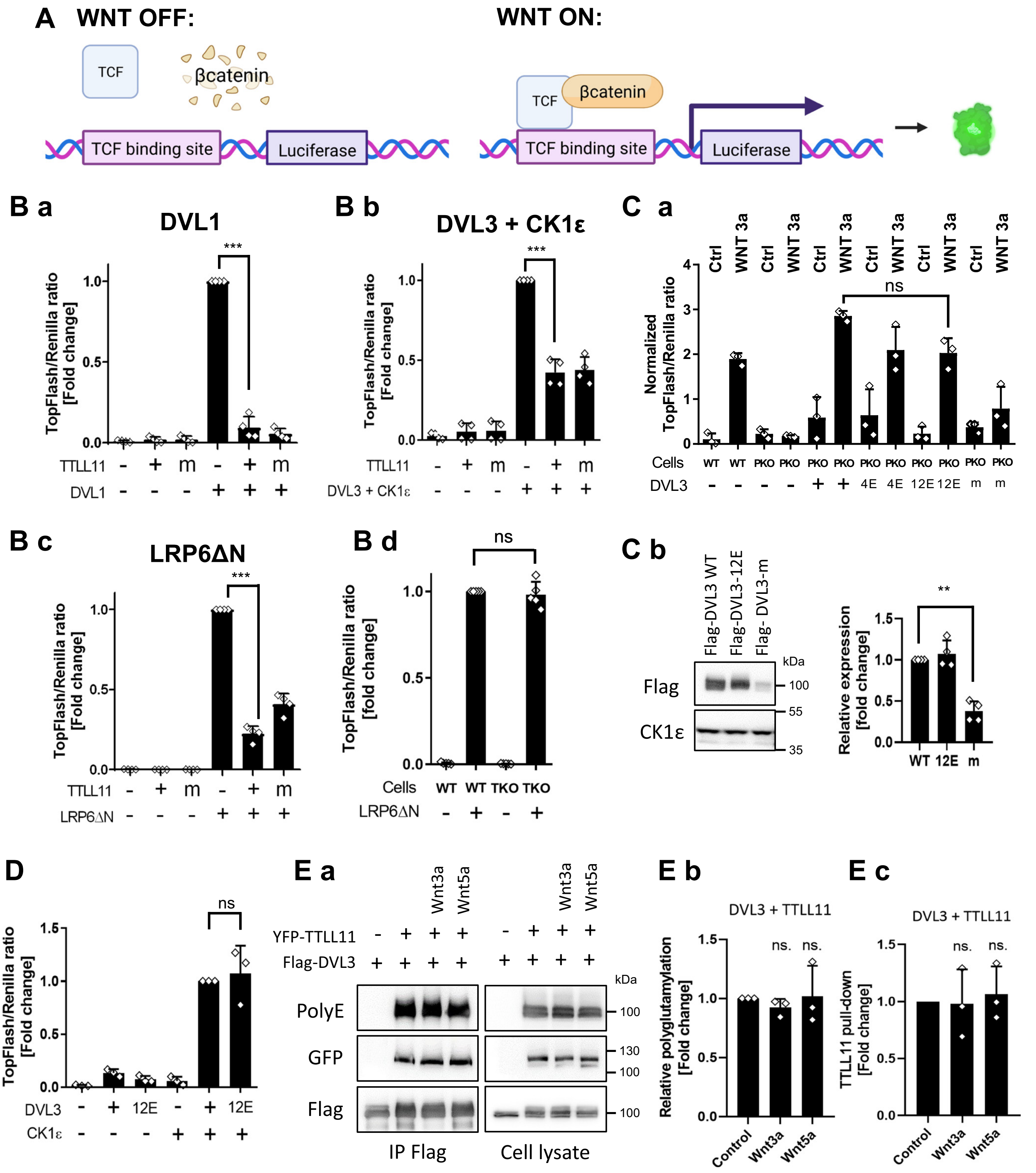
A WNT OFF:
TCF
βcatenin
TCF binding site
Luciferase
WNT ON:
TCF
βcatenin
TCF binding site
Luciferase
B a DVL1
TopFlash/Renilla ratio [Fold change]
TTLL11 - + m - + m
DVL1 - - - + + +
B b DVL3 + CK1ε
TopFlash/Renilla ratio [Fold change]
TTLL11 - + m - + m
DVL3 + CK1ε - - - + + +
C a
Normalized TopFlash/Renilla ratio
Ctrl WNT 3a Ctrl WNT 3a Ctrl WNT 3a Ctrl WNT 3a Ctrl WNT 3a Ctrl WNT 3a
ns
Cells WT WT PKO PKO PKO PKO PKO PKO PKO PKO PKO PKO
DVL3 - - - - + + 4E 4E 12E 12E m m
B c LRP6ΔN
TopFlash/Renilla ratio [Fold change]
TTLL11 - + m - + m
LRP6ΔN - - - + + +
B d
ns
TopFlash/Renilla ratio [fold change]
Cells WT WT TKO TKO
LRP6ΔN - + - +
C b
Flag-DVL3 WT
Flag-DVL3-12E
Flag- DVL3-m
kDa
Flag
CK1ε
100
55
35
Relative expression [fold change]
WT 12E m
D
ns
TopFlash/Renilla ratio [Fold change]
DVL3 - + 12E - + 12E
CK1ε - - - + + +
E a
Wnt3a Wnt5a
YFP-TTLL11 - + + +
Flag-DVL3 + + + +
PolyE
GFP
Flag
kDa
100
130
100
100
IP Flag
Cell lysate
E b DVL3 + TTLL11
ns. ns.
Relative polyglutamylation [Fold change]
Control Wnt3a Wnt5a
E c DVL3 + TTLL11
ns. ns.
TTLL11 pull-down [Fold change]
Control Wnt3a Wnt5a

 © The Author(s)

Figure EV4. DVL3 polyglutamylation does not affect activity in the Wnt/β-catenin pathway.

(**A**) TopFlash method scheme. WNT/β-catenin pathway activation results in induction of TCF target genes, and in production of active luciferase. (**B**) TopFlash reporter assay of TTLL11 and its inactive mutant E531G (indicated as m) with Wnt/β-catenin pathway activators: DVL1 (**Ba**), DVL3 + CK1 (**Bb**) and LRP6ΔN (**Bc**). (**Bd**) TopFlash reporter assay of LRP6ΔN in Hek293 T-rex (WT) or HEK293T-rex DVL1/TVL2/DVL3 KO cells (TKO). The data are represented as fold change compared to the inducer sample. *** represents (**Ba**) $P = 0.0001$; (**Bb**) $P = 0.0008$; (Bc) $P < 0.001$, ns not significant. (**C**) DVL3 rescue assays in HEK293T-rex RNF43/ZNRF3/DVL1/DVL2/DVL3 penta knockout cells (PKO) that lack endogenous DVL and cannot respond to Wnt3a. (**Ca**) PKO cells were transfected by indicated DVL3 variant and Wnt/β-catenin pathway signaling was induced by Wnt3a. WT cells = control. (**Cb**) DVL3m, DVL3-WT and DVL3-12E expression in cells transfected by the same amount of DNA. WB intensities are normalized to DVL3 WT; $n = 4$. CK1ε = loading control, $^{**}P = 0.002$ (**D**) Both DVL3 and DVL3-12E potently activate TopFlash assay upon CK1ε co-expression. (**Ea**) Analysis of DVL3 polyglutamylation and interaction with TTLL11 after stimulation by Wnt3a or Wnt5a. WB intensities for polyglutamylation (PolyE; **Eb**) or TTLL11 co-purification (GFP; **Ec**) were normalized to Flag signal (DVL3 amount). Results from biological three replicates are shown as a fold change to control polyglutamylation (**Eb**) or pulldown (**Ec**). TopFlash data represent mean ± SD; $n = 4$ for B; $n = 5$ for (**C**); $n = 3$ for (**D**), ns = not significant. Statistics: one-sample *t* test with theoretical mean = 1 for (**B**, **Cb**, **D**, **Eb**, **E**); one-way ANOVA with Dunnett' multiple comparisons test for Ca. (**B**, **D**, **E**) were performed in HEK293T cells. Source data are available online for this figure.

© The Author(s)

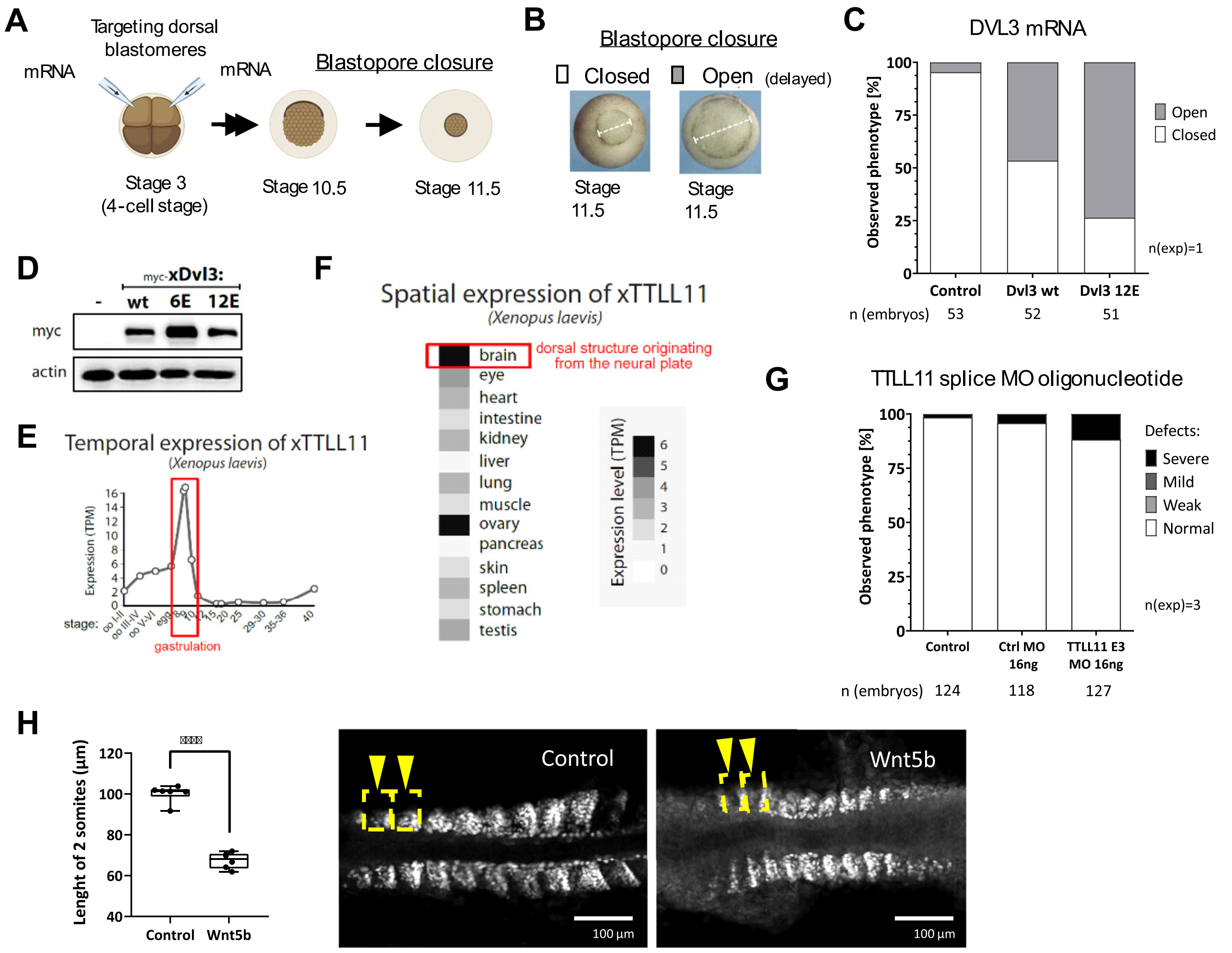

Figure EV5. Role of DVL3 polyglutamylation in *Xenopus laevis* and *Danio rerio* embryonal development.

(**A**) Both dorsal blastomeres of 4-cell *Xenopus laevis* embryo were injected with xDvl3 mRNA and embryos were observed during blastopore closure at stage 11.5. (**B**) Normal or delayed blastopore closure was assessed at stage 11.5. (**C**) Effect on uninjected embryos and embryos injected with either mRNA of xDvl3 WT or xDvl3-12E modification mimicking mutant. (**D**) WB analysis of xDvl3 protein amount in *Xenopus* embryo lysates. (**E**) xTTLL11 RNA expression during the development of *Xenopus laevis*. (**F**) Spatial expression of xTTLL11 in adult organism (**E**, **F** adapted from (Session et al, 2016)). (**G**) Effect of splicing MO targeting xTTLL11 exon 3 on gastrulation and neurulation (see also Fig. 5D). (**H**) The length of the first 2 anterior somites was measured for control embryo Wnt5b KO embryos ($n = 6$). Box plots are shown as median (middle bar) with 25th and 75th percentiles and whiskers showing min to max values. Statistical analysis was performed by unpaired *t* test; ****$P < 0.0001$. Source data are available online for this figure.

 © The Author(s)
